# Effects of Non‐Invasive Brain Stimulation for Degenerative Cerebellar Ataxia: A Systematic Review and Meta‐Analysis

**DOI:** 10.1002/mdc3.14205

**Published:** 2024-09-02

**Authors:** Akiyoshi Matsugi, Hiroyuki Ohtsuka, Kyota Bando, Yuki Kondo, Yutaka Kikuchi

**Affiliations:** ^1^ Faculty of Rehabilitation Shijonawate Gakuen University Daitō Japan; ^2^ Department of Physical Therapy Showa University School of Nursing and Rehabilitation Sciences Tokyo Japan; ^3^ National Center Hospital National Center of Neurology and Psychiatry Tokyo Japan; ^4^ Department of Rehabilitation for Intractable Neurological Disorders Institute of Brain and Blood Vessels Mihara Memorial Hospital Isesaki Japan

**Keywords:** cerebellum, ataxia, degenerative cerebellar ataxia, non‐invasive brain stimulation, repetitive transcranial magnetic stimulation, transcranial direct current stimulation

## Abstract

**Background:**

This systematic review and meta‐analysis aimed to assess the effectiveness of non‐invasive brain stimulation (NIBS), including repetitive transcranial magnetic stimulation (rTMS) and transcranial electrical stimulation (tES), as a neurological intervention for degenerative cerebellar ataxia (DCA) based on preregistration (PROSPERO: CRD42023379192).

**Objective:**

We aimed to explore clinical outcomes and examine the parameters associated with NIBS efficacy in DCA patients.

**Methods:**

The PubMed, Cochrane Library, CHINAL, and PEDro databases were searched for relevant randomized controlled trials (RCTs). Data extraction, quality assessment, and heterogeneity analyses were conducted; the Grading, Recommendations, Assessment, Development, and Evaluation was used to assess the quality of evidence and a meta‐analysis was performed.

**Results:**

Seventeen RCTs that included 661 patients on the scale for assessment and rating of ataxia (SARA) and 606 patients on the International Cooperative Ataxia Rating Scale (ICARS) were included. These RCTs showed a serious risk of bias (RoB) and low certainty of evidence for both outcomes. NIBS significantly reduced SARA (MD = −2.49, [95% confidence interval: −3.34, −1.64]) and ICARS (−5.27 [−7.06, −3.47]); the subgroup analysis showed significant effects: rTMS and tES reduced both outcomes. However, there were no significant differences in the effects of rTMS and tES. Additional subgroup analysis indicated the impact of rTMS frequency and the total number of tES sessions on ataxia.

**Conclusion:**

Non‐invasive brain stimulation may reduce ataxia in DCA patients, but the estimated effect size may change in future studies because the RoB was serious and the certainty of evidence was low, and the heterogeneity was high. To establish evidence for selecting NIBS methods and parameters, continued high‐quality RCTs are required.

Degenerative cerebellar ataxia (DCA) includes various neurodegenerative disorders characterized by progressive cerebellar dysfunction and Purkinje cell loss, leading to cerebellar atrophy.^1^ Degeneration of the cerebellum, brainstem, or spinal cord can induce diverse clinical symptoms. Limited treatment options affect daily activities and quality of life (QOL), highlighting the need for novel, safe, and effective non‐pharmacological interventions.^2^, ^3^ Physical neuro‐rehabilitation^4^, ^5^ and non‐invasive brain stimulation (NIBS),^6^ including repetitive transcranial magnetic stimulation (rTMS) and transcranial electrical stimulation (tES), have shown promise for treating cerebellar ataxia.

Repetitive transcranial magnetic stimulation can induce action potentials in focal cortical brain regions, affecting motor^7^ and sensory functions.^8^, ^9^ Its potential to induce long‐term changes in neural activity has made it a subject of interest for various neurological disorders,^10^ including cerebellar ataxia. Recent systematic reviews have reported the promising effects of rTMS on cerebellar ataxia symptoms in patients with stroke and DCA.^11^ In this systematic review,^11^ only two studies^12^, ^13^ have examined the effects of incorporated rTMS on the primary outcomes SARA and International Cooperative Ataxia Rating Scale (ICARS), and subgroup analyses examining the effects of stimulation parameters and disease severity were not available. Therefore, it remains unclear what parameter is effectiveness of rTMS on SARA and ICARS, and the effect of severity of disease on the primary outcome. Furthermore, heterogeneity in underlying conditions such as stroke and degenerative diseases can influence rTMS outcomes. Additionally, the number of studies examining factors affecting treatment efficacy in DCA, such as stimulus frequency and number of pulses, is insufficient.^7^, ^14^, ^15^ Similarly, tES, including transcranial direct current stimulation (tDCS), has shown clinical efficacy in DCA treatment^11^, ^16^ with current applied to the cerebellar cortex.^17^ These reviews considered each intervention independently and may have overlooked newer randomized controlled trials (RCTs).^18^ The dose and parameters of stimulation have not been fully investigated in previous SR, and the information needed for clinical use, for example, the effect of the amount of stimulation provided on SARA and ICARS, is still unclear. Based on these findings, we conducted a systematic review and meta‐analysis to fill these gaps and provide the latest findings on the effects of NIBS, including rTMS and tES, on cerebellar ataxia in patients with DCA.

Degenerative cerebellar ataxia includes heterogeneous disorders, such as autosomal dominant spinocerebellar ataxia,^19^ spinocerebellar ataxia (SCA),^20^, ^21^, ^22^ Friedreich's ataxia,^23^ multiple system atrophy with cerebellar ataxia (MSA‐C),^24^ and sporadic adult‐onset ataxia of unknown etiology.^25^ Therefore, in this systematic review, we did not limit our study to a single phenotype such as SCA. Furthermore, the present systematic review was limited to whether NIBS has an immediate effect of NIBS after intervention. Therefore, only effects observed immediately after the stimulus were included.

Randomized controlled trials and systematic reviews have examined the effects of NIBS on DCA; however, these studies have been insufficient to support clinical decision‐making. Uncertainties remain regarding the most effective stimulation conditions for rTMS, the number of tES sessions needed, disease severity, and outcomes. This study aimed to systematically review the collective data on NIBS, including both rTMS and tES, as a therapeutic intervention for cerebellar ataxia in patients with DCA. Our objectives were to assess the effect of rTMS and tES on ataxia severity, evaluate the stimulation parameters used in previous studies, and identify key considerations for future research. Additionally, we conducted subgroup analyses to compare the effects of rTMS and tES, and explore their safety and tolerability profiles in patients with DCA. This review aims to inform clinical practice and guide future research efforts in the management of DCA‐associated cerebellar ataxia.

## Methods

This systematic review was based on the Preferred Reporting Items for Systematic Reviews and Meta‐Analyses Protocols (PRISMA‐P) statement.^26^ This protocol was registered in the International Prospective Register of Systematic Reviews database (ID: 2023 CRD42023379192) and had been published as a protocol paper.^18^

### Eligibility Criteria

The inclusion criteria for this review were as follows: (1) RCTs, (2) patients underwent DCA, (3) rTMS or tES was used as an intervention, and (4) articles were written in English. The exclusion criteria were as follows: (1) study design was not an RCT and (2) reporting type was conference paper, protocol paper, or registration report.

### Information Sources and Search Strategy

PubMed, Cochrane Central Register of Controlled Trials, CINAHL, and PEDro databases were searched. The search was restricted to the English language and human participants. We created a search query for these search engines (Appendix S1). The search was conducted on July 20, 2023, and all articles published until this date were included.

### Selection Process

The search was performed by an independent reviewer (A.M.) using these databases, and four other reviewers confirmed the initial list of articles. Furthermore, manual searches were also conducted using relevant terms such as: “cerebellum,” “spinocerebellar degeneration,” “ataxia,” “transcranial magnetic stimulation,” “transcranial electrical stimulation,” and “clinical trial.” Rayyan (Cambridge, MA) and ENDNOTE 20 (Clarivate, Philadelphia, PA) were used to manage the studies in the databases.

### Data Collection Process

For each study, two independent reviewers screened the titles and abstracts and randomly assigned from a group of five (A.M., H.O., K.B., Y. Kondo, and Y. Kikuchi), to determine inclusion. Studies requiring full‐text evaluation were also assessed. The reviewer was blinded initially. Any inconsistent decisions were resolved by a third reviewer. Each pair was informed of the reviewer's identity during the final decision‐making process.

Two reviewers independently extracted data to obtain the study design, methodology, participant demographics, baseline characteristics, sample size, and effect measurements. Any discrepancies were resolved by a third reviewer. Authors were contacted for missing data. If the authors did not respond or refused to participate, only the available data were analyzed. The data were extracted using a Microsoft Excel spreadsheet.

### Data Items

We established and obtained the scale for assessment and rating of ataxia (SARA) and ICARS scores as primary outcomes, which were used to assess ataxic symptoms. We obtained 10‐ and 8‐m walk test (10MWT and 8MWT, respectively) data to examine the effect on gait speed, which reflects the severity of gait disturbance. The Berg Balance Scale (BBS) was used to estimate the severity of balance disorders associated with ataxia. The 9‐hole peg test (9HPT) was used to assess upper‐limb dexterity. The Functional Ambulator Category and Functional Independent Measure, which were obtained from the pro‐call articles, were not included in the collected articles. All outcomes were recorded immediately before and after the intervention period to examine the effects of NIBS.

### Study RoB Assessment

The risk of bias (RoB) was assessed using the Cochrane RoB tool (RoB 2.0).^27^ Two of five independent reviewers critically evaluated the included studies. The evaluation items included the following: (1) bias arising from randomization; (2) bias due to deviation from the intended intervention; (3) bias due to missing outcome data; (4) bias in the measurement of outcomes; and (5) bias in the selection of reported results. For each item, each study was evaluated as having a low, some concern, or high RoB. A judgment about the RoB arising from each domain was proposed using an algorithm based on answering the signaling questions.^27^ Any discrepancies were discussed and resolved by a third reviewer if necessary.

### Effect Measures and Synthesis Methods

SARA, ICARS, and BBS scores were obtained, and 10MWT, 8MWT, and 9HPT scores were obtained for time (s), mean of the pre‐post difference, and standard deviation (SD). The effect sizes were the mean difference (MD) and 95% confidence interval (CI) integrated using RevMan 5.4 (Cochrane Collaboration, Copenhagen, Denmark) for all outcomes.

In the case of missing statistics for each article, we first requested the author to provide the data. If data were provided in response to a request, they were used; otherwise, statistics were calculated using the method recommended by Cochrane and used in the meta‐analysis. Specifically, if the SD of the MD was not provided, we calculated the effect size of the MD and SD from the MD and SD before and after the intervention in other articles that were incorporated, and then determined the correlation coefficient between the MD and SD that were reported. This correlation coefficient was used to supplement the SDs of studies that did not report the SDs of the MD.

If more than two randomized (or quasi‐randomized) controlled trials reported the same outcomes, RevMan 5.4 software was used to calculate the weighted MD. Mean and SD were used for continuous data. Random‐effects models were used to obtain pooled estimates, and the results were described using forest plots in RevMan 5.4.

To examine the effects of NIBS, a meta‐analysis was conducted without separating the tES and rTMS groups. Subsequently, subgroup analysis was performed according to the type of intervention, which was divided into rTMS and tES. Furthermore, in rTMS, a subgroup analysis was performed based on frequency and not coil location because none of the studies stimulated anything other than the cerebellum. In tES, we performed a subgroup analysis based on the session number of tES and did not perform an analysis based on the type of polarity (anode or cathode), current (direct current, alternating current, or random noise), and intensity because there was no variation in the polarity or intensity applied and most of the direct current. Furthermore, to examine the effect of the baseline severity of ataxia, a subgroup analysis was performed according to the baseline SARA^28^ and ICARS^29^ scores in the different intervention conditions.

To estimate the dose effect of rTMS on SARA and ICARS scores, a meta‐regression analysis was conducted on the frequency and total pulse during the study. JASP software (version 0.17.1; University of Amsterdam, Amsterdam, Netherlands)^30^ was used for the meta‐regression analysis, with the alpha level set at 0.05.

Missing SD_change_ was defined as the SD of the MD and was calculated using formula (1), and the correlation coefficients (Corr) were calculated using formula (2) recommended by Cochrane, with the data in the article for which all data were reported fully sufficient.^31^, ^32^, ^33^ The average Corr 0.97 [range, 0.92–0.99] was used to calculate SD_change_ in individual studies with a lack of SD_change_.

(1) $${\mathrm{SD}}_{\text{change}}=\text{square}\left(\left[{{\mathrm{SD}}_{\text{baseline}}}^{2}+{{\mathrm{SD}}_{\text{final}}}^{2}\right]–\left[2\times \text{Corr}\times {\mathrm{SD}}_{\text{baseline}}\times {\mathrm{SD}}_{\text{final}}\right]\right)$$

(2) $$\text{Corr}\;=\begin{array}{l} \left({{\mathrm{SD}}_{\text{experiment, baseline}}}^{2}+{{\mathrm{SD}}_{\text{control, baseline}}}^{2}\right)/\\ (2\times {\mathrm{SD}}_{\text{experiment, baseline}}\times {\mathrm{SD}}_{\text{control, baseline}}) \end{array}$$

Sensitivity analysis was performed by changing the correlation coefficient to 0.5, estimating the effect size and examining whether the integrated value varied in the opposite direction.

### Reporting Bias Assessment

Funnel plots were used to determine publication bias.

### Certainty Assessment

The Grading, Recommendations, Assessment, Development, and Evaluation (GRADE) framework^34^ was used to evaluate the overall quality of evidence across all outcomes. The evaluation items included: (1) study design, (2) RoB, (3) inconsistency, (4) indirectness, (5) imprecision, and (6) other considerations. These indicators were used to determine the level of certainty associated with the estimated effect, categorized as “very low,” “low,” “moderate,” or “high.”

## Results

### Study Selection

**Figure 1:**
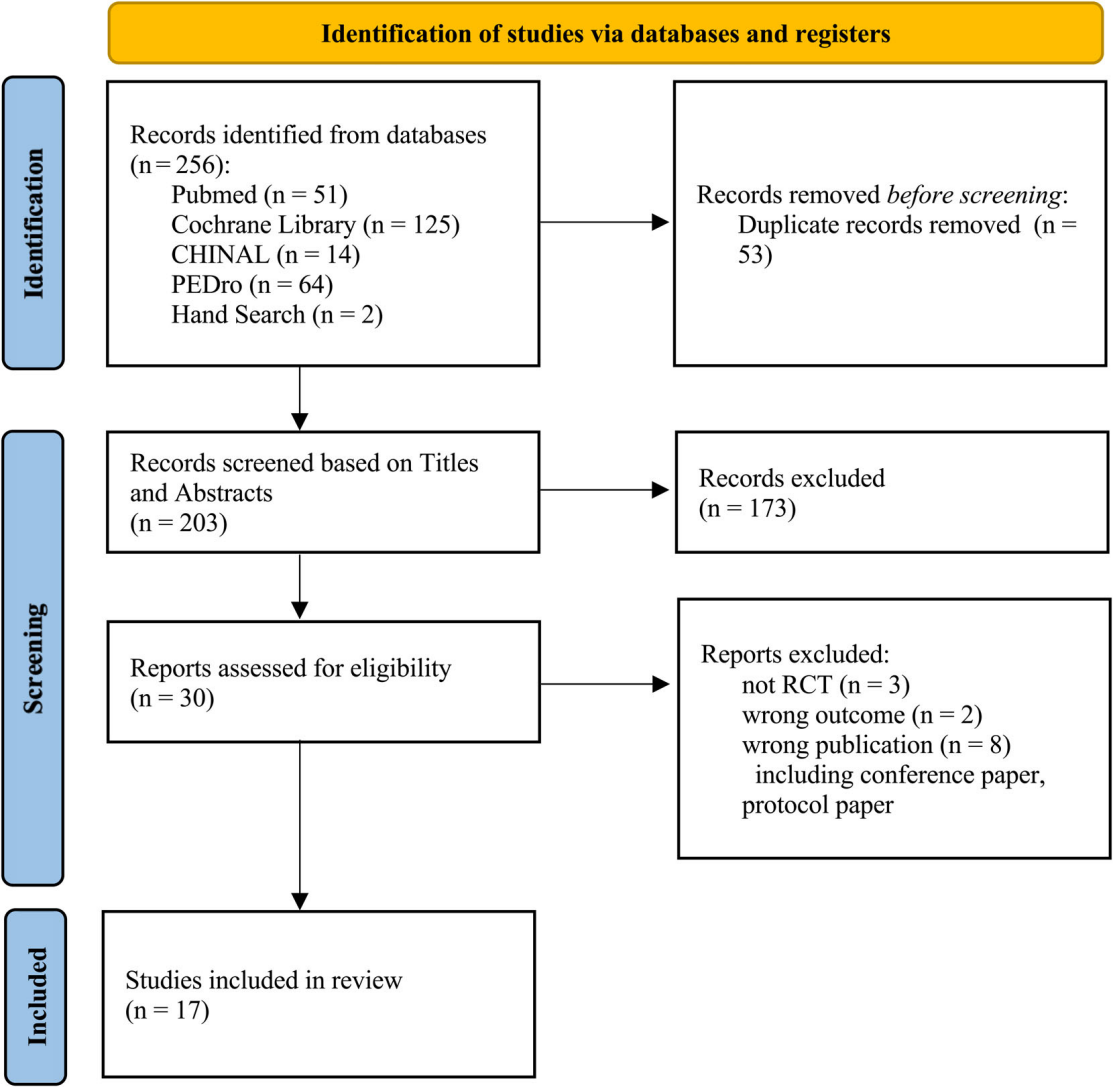
Flowchart of the search strategy in the Preferred Reporting Items for Systematic Reviews and Meta‐Analyses (PRISMA) flow diagram. This flowchart outlines the stages of article identification and selection, which led to the inclusion of 17 articles that met the criteria for review.

A total of 256 articles were retrieved using a database search and additional records. After eliminating duplicates, the titles and abstracts of 203 publications were selected. Among these, 30 articles underwent full‐text screening for eligibility, and 13 articles were excluded based on the following criteria: (1) non‐RCT (n = 3); (2) the outcome measure did not include symptoms associated with cerebellar ataxia (n = 2); and (3) the publication was not an original research paper, such as a conference paper or protocol paper (n = 8). Finally, 17 articles^12^, ^13^, ^31^, ^32^, ^33^, ^35^, ^36^, ^37^, ^38^, ^39^, ^40^, ^41^, ^42^, ^43^, ^44^, ^45^, ^46^ met the inclusion criteria and were included in the meta‐analysis. A flowchart illustrating the selection process is shown in Figure 1. The review process was documented using a checklist of the PRISMA Items for Systematic Reviews and Meta‐Analyses (Appendix S2).

### Study Characteristics

#### Overall

The characteristics of the studies identified using rTMS and tES are shown in Tables S1 and S2, respectively. A total of 563 patients (354 in the rTMS group and 209 in the tES group) were included in the 17 studies. The most common SCA subtype was SCA3; only MSA‐C was included in this subtype. Three studies^39^, ^40^, ^45^ did not report any funding.

#### Characteristics of rTMS and tES Methodology

Eight studies included an rTMS group. Only one study had a crossover design^12^; the others had a parallel‐group design. The stimulation site was the cerebellum in all studies.^12^, ^13^, ^32^, ^33^, ^41^, ^44^, ^45^, ^46^ Circle,^33^, ^44^, ^45^ figure‐of‐eight,^32^ and double‐cone coils^12^, ^46^ were used for active stimulation. The stimulation intensity was set to 80% resting motor threshold (rMT),^13^ 90% rMT,^12^ 100% rMT,^32^, ^33^ 100% motor threshold (MT),^46^ and 250% rMT.^45^ The method of determining rMT was reported in only two studies,^45^, ^46^ and there was a lack of information, such as stimulus site or EMG‐recorded muscle information, in other studies. A placebo coil was used for sham stimulation in three studies.^12^, ^13^, ^32^ We were unable to find reports on whether sham electrical stimulation was used in any study. The frequency was set at 0.17 Hz,^44^, ^45^ 1 Hz,^12^, ^32^, ^33^, ^41^ 10 Hz,^46^ and 50 Hz (for TBS).^13^, ^32^ The total pulses per day were set at 30–2400 pulses, and the total pulses during the study were set at 630–27,000 pulses. The intervention lasted 5–21 days.

There were nine tES studies,^31^, ^35^, ^36^, ^37^, ^38^, ^39^, ^40^, ^42^, ^43^ targeting the cerebellum^31^, ^37^, ^38^, ^39^, ^40^, ^42^, ^43^ or motor cortex.^35^, ^36^ The intensity was set at 2 mA,^35^, ^36^, ^37^, ^38^, ^39^, ^40^, ^42^, ^43^ 3 mA,^42^ or 3.3 mA.^31^ The duration of the intervention (stimulation) per session was set at 20 min,^31^, ^35^, ^38^, ^39^, ^40^, ^42^ 30 min,^37^ 40 min,^36^ or 60 min.^42^ The number of sessions for the study was set at 1,^35^, ^40^, ^42^ 5,^31^, ^36^ or 10.^37^, ^38^, ^39^, ^43^

### RoB in Studies

**Figure 2:**
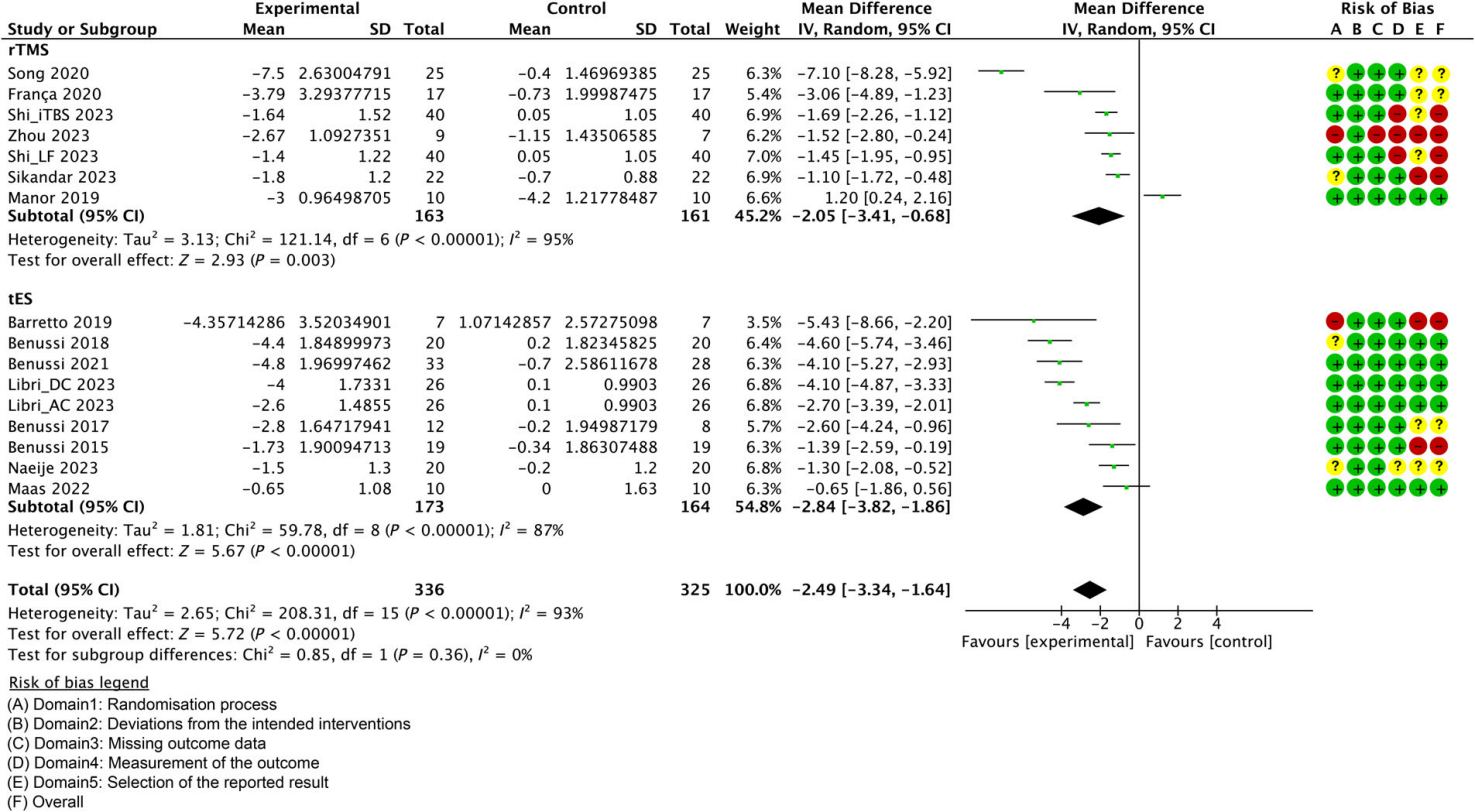
Forest plot analysis and risk of bias (RoB) based on the scale for assessment and rating of ataxia (SARA). “–” indicates “high RoB,” “?” indicate “some concerns,” and “+” indicates “low RoB.” rTMS, repetitive transcranial magnetic stimulation; tES, transcranial electrical stimulation.

**Figure 3:**
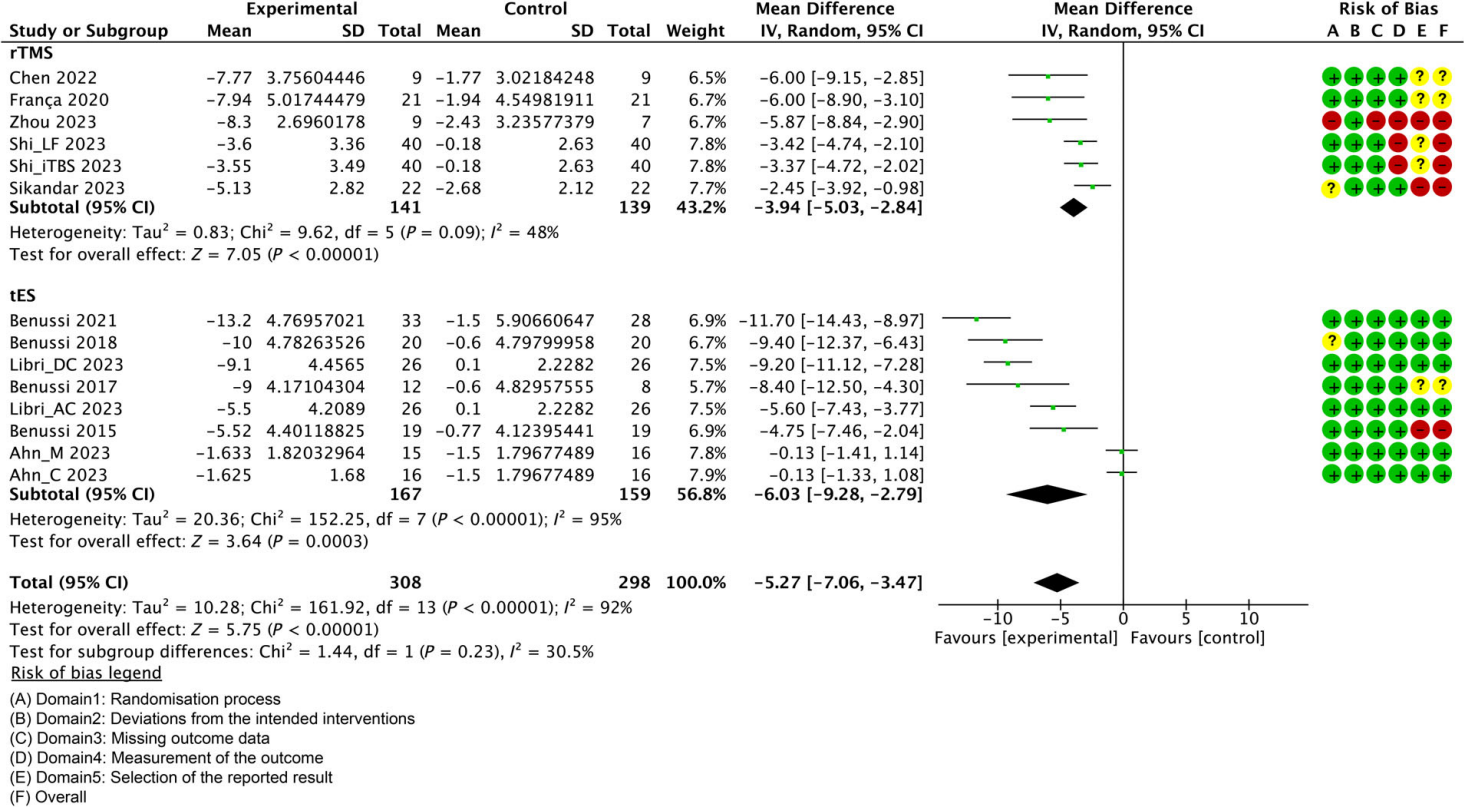
Forest plot analysis and risk of bias (RoB) based on the International Cooperative Ataxia Rating Scale (ICARS). “–” indicates “high RoB,” “?” indicate “some concerns,” and “+” indicates “low RoB.” rTMS, repetitive transcranial magnetic stimulation; tES, transcranial electrical stimulation.

Figures 2 and 3 and Figures S1 to S3 indicate the RoB for SARA, ICARS, BBS, gait speed, and 9HPT. Figure S4 shows the percentages of studies in the six domains and overall bias. The overall RoB was approximately 70%. However, for RoB of “selection of the reported result,” the high risk was >30% and the low risk was <40%.

Some RoBs of the overall domain were judged “high.” In an rTMS study, Shi et al^32^ reported an outcome that was different from preregistration and blinding may have been incomplete. Sikandar et al^33^ reported outcomes that were different from preregistration, and some outcomes were obtained in the same domain. A careful review of the report by Zhou et al^46^ suggests that the method of randomization for allocation and concealment is inadequate. The blinding may have been incomplete for the assessor, and 20% of the subjects dropped out, but the reason for this was not clearly stated.^46^ Furthermore, Zhou et al^46^ reported that only SARA was planned to be acquired in registration, but ICARS was also reported as a result. In tES, a study reported in 2015 was not registered, and it was not possible to verify whether the study was conducted according to predefined outcomes and analysis methods.^40^

### Results of Syntheses

When there were not enough data on the SD_change_ of MD (pre‐post) in the original report, we requested the data from the corresponding authors. The authors of two studies^35^, ^43^ gave the requested data. One group^45^ responded to our request, but missed the data used in the original report. There were no responses to our request for eight studies.^13^, ^37^, ^38^, ^39^, ^40^, ^41^, ^44^, ^46^ Two groups^12^, ^36^ reported all individual data used in the original report; therefore, we calculated MDs and SDs using individual data from patients with SCA and MSA‐C.

Tables 1 and 2 indicate the GRADE quality of the evidence for the primary (Table 1) and secondary (Table 2) outcomes, and the RoBs in these outcomes were “serious.”

**TABLE 1:** Grading of Recommendations Assessment, Development, and Evaluation (GRADE) quality of primary outcomes; assessment and rating of ataxia (SARA) and International Cooperative Ataxia Rating Scale (ICARS)

Outcome	Certainty assessment							No. of patients		Effect		
	No. of studies	Study design	Risk of bias	Inconsistency	Indirectness	Imprecision	Other considerations	Active‐NIBS	Sham‐NIBS	Absolute (95% CI)	Certainty	Importance
SARA	16	Randomized trials	Serious	Serious^a^	Not serious	Not serious	Publication bias strongly suspected dose response gradient^b^	336	325	MD 2.49 points lower (3.34 lower to 1.64 lower)	⨁⨁◯◯ Low	Critical
ICARS	14	Randomized trials	Serious	Serious^a^	Not serious	Not serious	Publication bias strongly suspected dose response gradient^b^	308	298	MD 5.27 points lower (7.06 lower to 3.47 lower)	⨁⨁◯◯ Low	Critical

^a^ *I* ^2^ > 90%.

^b^ Suggested by funnel plot.

CI, confidence interval; MD, mean difference; NIBS, non‐invasive brain stimulation.

**TABLE 2:** Grading of Recommendations Assessment, Development, and Evaluation (GRADE) quality of primary outcome

Outcome	Certainty assessment							No. of patients		Effect	Certainty	Importance
	No. of studies	Study design	Risk of bias	Inconsistency	Indirectness	Imprecision	Other considerations	Active‐NIBS	Sham‐NIBS	Absolute (95% CI)		
BBS	1	Randomized trials	Serious	Serious^a^	Not serious	Serious^b^	None	22	22	MD 3.23 points higher (1.95 higher to 4.51 higher)	⨁◯◯◯ Very low	Important
10MWT	3	Randomized trials	Serious	Not serious	Not serious	Serious^b^	None	115	115	MD 0.66 seconds lower (1.68 lower to 0.36 higher)	⨁⨁◯◯ Low	Important
8MWT	4	Randomized trials	Not serious	Not serious	Not serious	Serious^b^	None	61	57	MD 0.84 second lower (1.72 lower to 0.04 higher)	⨁⨁⨁◯ Moderate	Important
9HPT (dominant)	5	Randomized trials	Serious	Not serious	Not serious	Serious^b^	None	122	118	MD 1.84 seconds lower (4.99 lower to 1.3 higher)	⨁⨁◯◯ Low	Important
9HPT (not dominant)	5	Randomized trials	Serious	Not serious	Not serious	Serious^b^	None	122	118	MD 2.54 seconds lower (4.46 lower to 0.61 lower)	⨁⨁◯◯ Low	Important
9HPT (not separate)	2	Randomized trials	Serious	Not serious	Not serious	Serious^b^	None	29	29	MD 1.87 seconds lower (3.76 lower to 0.02 higher)	⨁⨁◯◯ Low	Important

^a^ Only one study was included.

^b^ The sample size was too small (n < 400).

10MWT, 10 meter walk test; 8MWT, 8 meter walk test; 9HPT, 9 hole peg test; BBS, Berg Balance Scale; CI, confidence interval; MD, mean difference; NIBS, non‐invasive brain stimulation.

Individual studies and their effect sizes are shown in Figures 2 and 3 and Figures S1 to S3. Overall, the NIBS intervention had a beneficial effect on SARA (MD = −2.49, 95% CI [−3.34 to −1.64], *z* = 5.72, *P* < 0.00001) and ICARS (MD = −5.27, 95% CI [−7.06 to −3.47], *z* = 5.75, *P* < 0.00001) scores. Regarding secondary outcomes, only one study included the BBS (MD = 3.23, 95% CI [1.95, 4.51], *z* = 4.96, *P* < 0.00001). There was a beneficial effect on 9HPT (not separated) (MD = −1.87, 95% CI [−3.76 to 0.02], *z* = 1.94, *P* = 0.05). Meanwhile, there was no beneficial or harmful effect on 10MWT (MD = −0.66, 95% CI [−1.68 to 0.36], *z* = 1.27, *P* = 0.21), 8MWT (MD = −0.84, 95% CI [−1.72 to 0.04], *z* = 1.88, *P* = 0.06), 9HPT (dominant hand) (MD = −1.84, 95% CI [−4.99 to 1.3], *z* = 1.15, *P* = 0.25), or 9HPT (not‐dominant hand) (MD = −2.54, 95% CI [−4.46 to −0.61], *z* = 2.58, *P* = 0.01).

In contrast, *I* ^2^, a statistic that indicates the level of heterogeneity of the overall effect of NIBS on all outcomes, was >70% without 9HPT (not separated). For the primary outcome, the *I* ^2^ values of rTMS for the SARA and ICARS were 95% and 87%, respectively. The *I* ^2^ values in the tES for the SARA and ICARS were 48% and 95%, respectively.

Due to the high heterogeneity of the main outcomes, we performed subgroup analyses to explore these factors. rTMS was analyzed for stimulus frequency and number of stimuli. When the stimulus frequency was categorized as very low (<1 Hz), low (1 Hz), middle (10 Hz), or high (50 Hz), SARA showed significant differences between groups (χ^2^ = 23.8, df = 3, *P* < 0.0001, *I* ^2^ = 87.4%), and significant improvement was found in the low and middle groups (Figure S5). In contrast, in the ICARS, all groups showed significant improvements, with no significant difference between groups (χ^2^ = 2.28, df = 2, *P* = 0.32, *I* ^2^ = 12.1%) (Figure S6). The number of stimuli was classified as low‐dose (<10,000), middle‐dose (10,000–20,000), or high‐dose (>20,000). There were no significant differences between subgroups in either SARA (χ^2^ = 2.28, df = 2, *P* = 0.32, *I* ^2^ = 12.1%) or ICARS (χ^2^ = 2.28, df = 2, *P* = 0.32, *I* ^2^ = 12.1%). It should be noted that the low‐dose SARA group did not show any significant improvement. Furthermore, we examined the correlation between the main outcome and the frequency and number of stimuli using a meta‐regression analysis, which is also included in PRISMA. Figure S7 and Tables S3 and S4 show the results of the meta‐regression analysis of the frequency and number of pulses during the study. In SARA, the frequency was significantly affected (*z* = −2.126, *P* = 0.033 [95% CI, −0.123 to −0.005]), but the total pulse was not (*z* = −0.794, *P* = 0.427 [95% CI, −2.073 × 10^−4^ to 8.778 × 10^−5^]). In ICARS, there was no significant effect of frequency (*z* = 0.586, *P* = 0.586 [95% CI, −6.64, −2.271]) or total pulse (*z* = 0.756, *P* = 0.45 [95% CI, −6.538 × 10^−5^ to 1.474 × 10^−4^]).

In tES, when the number of sessions was categorized as low‐dose (1 session), middle‐dose (5 sessions), or high‐dose (10 sessions), SARA did not show significant differences between groups (χ^2^ = 0.04, df = 2, *P* = 0.98, *I* ^2^ = 0%), but there were significant differences in ICARS (χ^2^ = 61.23, df = 1, *P* < 0.00001, *I* ^2^ = 98.4%). Because a moderate dose was lacking, a comparison was conducted between the low‐ and high‐dose groups (Figure S8).

In baseline severity of ataxia based on SARA, when the severity of ataxia was categorized as severe (>14.25), moderate (10–14.25), mild (5.5–10), or minimal activities‐of‐daily‐living dependency (<5.5), SARA in rTMS showed a significant difference between groups (χ^2^ = 4.13, df = 1, *P* = 0.04, *I* ^2^ = 75.8%) whereas SARA in tES did not (χ^2^ = 0.37, df = 2, *P* = 0.83, *I* ^2^ = 0%). In baseline severity of ataxia based on ICARS, when the severity of ataxia was categorized as equivalent degree of disease severity Stage 3 (>55.9), stage 2 (37.5–55.9), stage 1 (15.45–37.5), or stage 0 (<15.45), ICARS in rTMS (χ^2^ = 0.98, df = 1, *P* = 0.32, *I* ^2^ = 0%) and in tES (χ^2^ = 3.44, df = 1, *P* = 0.06, *I* ^2^ = 70.9%) did not show a significant difference (Figures S9 and S10).

Sensitivity analysis examined whether changing the correlation coefficient to 0.5 would change the results of the effect estimates. As shown in Figures S11 and S12, the integrated MDs were −2.23 [95% CI, −2.97 to −1.49] for SARA, −4.42 [95% CI, −6.12 to −2.17] for ICARS, −0.26 [95% CI, −0.79 to 0.27] for 10MWT, 0.12 [95% CI, −0.6 to 0.85] for 8MWT, 0.19 [95% CI, −1.36 to 1.74] for 9HPT (dominant hand), −1.83 [95% CI, −3.1 to −0.55] for 9HPT (not dominant hand), and −1.81 [95% CI, −8.82 to 5.19] for 9HPT (not separated). These estimates did not differ significantly from the direction of the effect estimates presented in Figures 2 and 3 and Figures S1 to S3.

### Reporting Biases

Publication bias was strongly suspected based on funnel plots for the SARA, ICARS, 10MWT, 8MWT, and 9HPT (Figure S13).

### Certainty of Evidence

Grading, Recommendations, Assessment, Development, and Evaluation quality of the evidence of primary outcomes was judged as follows: SARA is “low,” ICASRS is “low,” BBS is “very low,” 10MWT is “low,” 8MWT is “moderate,” and 9HPTs in dominant, not‐dominant, and not separate are “low” (Tables 1 and 2).

In the primary outcomes, the RoB was “serious,” inconsistency was “serious” with the reason of *I* ^2^ > 90%, and publication bias was strongly suspected as the reason for the obvious asymmetry of the funnel plot in both outcomes. In SARA, subgroup and meta‐regression analyses suggested a dose–response gradient. Based on these findings, the certainty of evidence was judged as “low” in SARA and “very low” in ICARS. In the secondary outcomes, imprecision was “serious” in all outcomes owing to the small sample size (n < 400). The inconsistency was “serious” only in BBS because only one study was included.

## Conclusions

We conducted a systematic review and meta‐analysis to determine whether NIBS reduces ataxia in patients with DCA. Seventeen RCTs were incorporated and meta‐analyzed, indicating that NIBS may reduce ataxia, as assessed by SARA and ICARS; however, the RoB was high and the certainty of evidence was considered low. Therefore, caution should be exercised when using this evidence in clinical practice and the expected effect may change depending on future studies.

The primary question was whether NIBS immediately improved ataxia. The meta‐analysis revealed significant improvements in SARA and ICARS scores, consistent with the results of independent studies. Secondary outcomes related to ataxia showed that only one study reported significant improvements in the BBS and 9HPT (non‐dominant hand). This suggests that NIBS does not improve gait speed or dominant hand dexterity. Notably, the SARA and ICARS indices of ataxia are comprehensive indices that include gait ability and upper‐extremity ataxia. However, the effects cannot be observed for single items, and more detailed studies are needed to determine which NIBS items improve.

The key question was whether rTMS or tES is more effective. The subgroup analysis showed that rTMS and tES did not differ significantly in their effects on the SARA and ICARS scores. This suggested that there was no difference between the expected effects of rTMS and tES on ataxia. However, only the effect of tES on the ICARS was moderately heterogeneous (*I* ^2^ = 48%), with a consistent direction in effect, which may explain its use in clinical practice. Further, this review aimed to examine the consistency of stimulation methods and parameters. Tables S1 and S2 show the inconsistencies for both rTMS and tES, indicating the need for continued examination of protocol settings that will contribute to improvement.

The need to control the stimulation method has been highlighted for rTMS. Inconsistencies were observed in the type of coil, stimulation frequency, number of stimulations, and number of stimulation locations. Furthermore, the rMT measurement methods used to set the stimulation intensity were not clearly stated, and in many cases, the sham stimulation method was not described at a reproducible level. Regarding frequency, two studies employing 0.17 Hz showed no significant effect, and significant effects appeared at 1 Hz or higher. The meta‐regression analysis showed a positive correlation with greater effects on SARA as the frequency increased. Applying at least 1 Hz, and preferably 10 or 50 Hz, may be better. However, the number of pulses applied during the intervention period in the study that applied 0.17 Hz was only approximately 600, which may have been influenced by the fact that the number of pulses was very small (1/10 to 1/900 in other studies). Furthermore, it should be noted that an intermittent stimulation pattern^47^ was used at 50 Hz, and this difference in pattern may have caused the difference in effectiveness.

The effects of rTMS depend on the frequency and stimulation pattern. In general, frequencies <1 Hz have been reported to decrease excitability in brain regions and suppress cerebellar inhibition (CBI)^6^, ^48^ or inhibit learning^49^ when applied to the cerebellum. By contrast, frequencies above 10 Hz are believed to increase the excitability of the brain regions to which they are applied. Of the studies included in this study, six^12^, ^32^, ^33^, ^41^, ^44^, ^45^ used low‐frequency stimulation (≤1 Hz) and three^13^, ^32^, ^46^ used middle (>10 Hz) or high‐frequency (50 Hz) stimulation. However, they did not show antagonistic effects, such as worsening of symptoms, under either stimulation conditions. This hinders our understanding of the mechanisms by which rTMS alleviates cerebellar ataxia.

For tES, most studies used direct current; one used alternating current.^42^ One study compared direct and alternating current, showing no differences in effectiveness.^42^ More studies are needed to determine the superior comparison method. Subgroup analysis showed that a higher number of sessions was associated with greater improvement in response to ICARS. This finding is useful for clinical interventions. Meanwhile, regarding probes for tES, many of the included tDCS studies chose to stimulate the left and right cerebellar hemispheres simultaneously and extensively using a wide probe. This broad stimulation potential may be advantageous over rTMS, which is a localized stimulation method.

The severity of ataxia may affect the efficacy of NIBS. The subgroup analysis based on baseline severity indicated that rTMS may lead to greater improvements in more severe SARA scores. However, many RCTs involving milder and more severe cases are required to investigate the impact of disease severity.

Whether the results of this meta‐analysis should be adopted requires comparison with other evidence. Several systematic reviews and meta‐analyses have reported the effects of cerebellar rTMS^50^ and tES on cerebellar ataxia in stroke patients.^11^, ^51^ According to these reports, the number of included studies was large; however, the heterogeneity was low and there was some confidence in the effects of SARA and ICARS. The direction of the effect of NIBS on cerebellar ataxia is generally considered positive because the small number of studies included in this systematic review is believed to have increased heterogeneity, owing to the exclusion of a small number of studies that were outside the scope of the study. In addition, considering its clinical importance, promising treatment methods have not been established for DCA, and the effects of medications are limited. Although rehabilitation has been reported to be effective, the effect size is small and other low‐risk interventions are urgently needed. Since NIBS does not cause serious adverse events and can be used at low risk, it is considered of great clinical significance.

In the sensitivity analysis, the correlation coefficient between SD pre‐ and post‐intervention by calculating the missing SD was lowered from the calculated 0.97 to 0.5 to determine whether the direction of the effect estimate would change. The results did not show any significant changes in any of the outcomes. This indicates the reliability of the calculated effect estimates.

The safety of rTMS and tES was evaluated, and no serious adverse events were observed. Nine rTMS participants experienced mild side effects (discomfort, headaches, and leg pain) in both active and sham conditions. Patients with tES reported mild side effects (headache, pain, tingling, itching, burning, and drowsiness). NIBS had no serious long‐lasting adverse events, making it worthy of consideration.

The study had a few limitations. First, the number of studies was very small. We collected NIBS RCTs, but found only eight for rTMS and nine for tES. Of the articles published in 2023, when this review was planned, 38% were rTMS cases and 33% were tES cases, indicating recent research activity. Although the BBS, a crucial outcome of balance ability, was examined, only one study addressed it, limiting the effect estimation. Publication bias may have affected the primary outcomes, possibly leading to unpublished negative data. Second, the unavailability of data from the authors hindered accurate effect estimates. Data should be published in a reusable format. Third, this study had a limited examination of the long‐term effects, focusing mainly on immediate post‐intervention effects. There was one study^44^ on rTMS and three on tES^38^, ^39^, ^43^ that measured long‐term outcomes, but none adjusted for factors affecting SARA or ICARS, such as physical activity and dose of physical rehabilitation.^4^ Future studies should integrate outcome measures over time, considering the timing of measurements for long‐term effects by controlling for influential confounding factors, which varied in many studies. Fourth, we did not focus on outcomes other than motor‐related outcomes; therefore, patient‐reported outcome measures (PROM), QOL, cognitive function, and non‐motor symptoms were estimated using the Inventory of Non‐Ataxia Signs. In particular, it is possible that the effects on PROM and QOL were independent of the SARA and ICARS. Therefore, future systematic reviews should focus on these outcomes.

The mechanism underlying the improvement of ataxia using NIBS remains unclear. CBI reflects the function of cerebellar output to the contralateral M1^52^ and indicates the severity of ataxia.^53^ In further study, the functionality of cerebellar output, estimated by CBI, may be useful in predicting responders to NIBS. Other possible mechanisms include the neuroprotection effect^54^ and increased cerebellar reserve.^55^ Clarifying the mechanism of the effect and the factors affecting responders are also important future research questions.

Despite several concerns, the results of this study may provide evidence for selecting NIBS in clinical practice. Whether rTMS or tES is more effective or the optimal stimulation parameters remain unclear. tES may be superior to rTMS in terms of medical economics due to fewer side effects and lower equipment costs. These points need to be compared and investigated continuously.

In conclusion, we conducted a systematic review to estimate the effect of NIBS on cerebellar ataxia in patients with DCA based on the latest RCT findings. NIBS may reduce ataxia; however, the estimated effect size may change in future studies because the RoB of the included studies was serious, the certainty of evidence was low, and the heterogeneity was high. High‐quality RCTs are required to establish evidence for NIBS as a treatment option.

## Author Roles

(1) Research Project: A. Conception, B. Organization, C. Execution; (2) Statistical Analysis: A. Design, B. Execution, C. Review and Critique; (3) Manuscript: A. Writing of the First Draft, B. Review and Critique.

A.M.: 1A, 1B, 1C, 2A, 2B, 2C, 3A, 3B.

H.O.: 1A, 1C, 2A, 2B, 2C, 3B.

K.B.: 1A, 1C, 2C, 3B.

Y.Kondo: 1A, 1C, 2C, 3B.

Y.Kikuchi: 1A, 1C, 2C, 3B.

## Disclosures

**Ethical Compliance Statement:** We confirm that we have read the Journal's position on issues involved in ethical publication and affirm that this work is consistent with those guidelines. Institutional review board approval and patient consent were not required for this study. We have familiarized ourselves with the Journal's ethical publication guidelines and affirm that this work adheres to those principles.

**Funding Sources and Conflicts of Interest:** This study was supported by the Japan Society for the Promotion of Science (JSPS) KAKENHI (grant number 23K10418) and the Institute of Health Sciences at Shijonawate Gakuen University (grant number IHSS2301). The authors have no conflicts of interest to declare.

**Financial Disclosures for the Previous 12 Months:** The authors declare that there are no additional disclosures to report.

## Supporting information


**Appendix S1.** Searching strategy.

**Appendix S2.** PRISMA checklist.

**Figure S1.** Forest plot analysis and risk of bias (RoB) based on the Berg Balance Scale (BBS). “–” indicates “high RoB,” “?” indicate “some concerns,” and “+” indicates “low RoB.”

**Figure S2.** Forest plot analysis and risk of bias (RoB) based on gait speed (10MWT and 8MWT). “–” indicates “high RoB,” “?” indicate “some concerns,” and “+” indicates “low RoB.” 10MWT, 10 meter walk test; 8MWT, 8 meter walk test.

**Figure S3.** Forest plot analysis and risk of bias (RoB) based on the 9 hole peg test (9HPT) in (a) dominant hand, (b) not dominant hand and (c) not separated measure. “–” indicates “high RoB,” “?” indicate “some concerns,” and “+” indicates “low RoB.” rTMS, repetitive transcranial magnetic stimulation; tES, transcranial electrical stimulation.

**Figure S4.** Percentage of number of studies about risk of bias (RoB) in intention to treat. Horizontal scale indicates percentage of number of studies. Vertical categories indicate the domain of RoB.

**Figure S5.** Forest plot for subgroup analysis regarding (a) frequency (Hz) and (b) dose (total pulse during study) of rTMS on SARA. rTMS, repetitive transcranial magnetic stimulation; SARA, assessment and rating of ataxia.

**Figure S6.** Forest plot for subgroup analysis regarding (a) frequency (Hz) and (b) dose (total pulse during study) of rTMS on ICARS. rTMS, repetitive transcranial magnetic stimulation; ICARS, International Cooperative Ataxia Rating Scale.

**Figure S7.** Meta‐regression analysis on primary outcomes. The vertical scales indicate mean difference (MD) of outcome and horizontal scales indicate frequency (a and c) and total pulse during study (b and d). The center of circle indicates the MD, and size of circle indicate 95% confidence interval (CI). The dots lines indicate liner regression line. rTMS, repetitive transcranial magnetic stimulation; SARA, assessment and rating of ataxia; ICARS, International Cooperative Ataxia Rating Scale.

**Figure S8.** Forest plot for subgroup analysis regarding dose (number of session) of tES on. (a) SARA and (b) ICARS. SARA, scale for assessment and rating of ataxia; tES, transcranial electrical stimulation.

**Figure S9.** Forest plot for subgroup analysis regarding baseline severity in rTMS on (a) SARA and (b) ICARS. SARA, scale for assessment and rating of ataxia; tES, transcranial electrical stimulation.

**Figure S10.** Forest plot for subgroup analysis regarding baseline severity in tES on (a) SARA and (b) ICARS. SARA, scale for assessment and rating of ataxia; tES, transcranial electrical stimulation.

**Figure S11.** Forest plot for sensitivity analysis for primary outcomes. SARA, scale for assessment and rating of ataxia; ICARS, International Cooperative Ataxia Rating Scale; rTMS, repetitive transcranial magnetic stimulation; tES, transcranial electrical stimulation.

**Figure S12.** Forest plot for sensitivity analysis for secondary outcomes. 10MWT, 10 meter walk test; 8MWT, 8 meter walk test; 9HPT, 9 hole peg test; rTMS, repetitive transcranial magnetic stimulation; tES, transcranial electrical stimulation.

**Figure S13.** Funnel plot of NIBS. NIBS, non‐invasive brain stimulation; SARA, scale for assessment and rating of ataxia; ICARS, International Cooperative Ataxia Rating Scale; 10MWT, 10 meter walk test; 8MWT, 8 meter walk test; 9HPT, 9 hole peg test; rTMS, repetitive transcranial magnetic stimulation; tES, transcranial electrical stimulation.

**TABLE S1.** Characteristics of the studies included in the rTMS studies. RCT, randomized control trial; E, experimental; C, control; SCD, spinocerebellar degeneration; SCA, spinocerebellar ataxia; MSA, multiple system atrophy; rTMS, repetitive transcranial magnetic stimulation; iTBS, intermittent theta burst stimulation; rMT, resting motor threshold; MT, motor threshold; M1, primary motor cortex; FDI, first dorsal interosseous muscle; APB, abductor pollicis brevis muscle; 10MWT, 10 meter walk test; SARA, scale for assessment and rating of ataxia; 9HPT, 9 hole peg test; ICARS, International Cooperative Ataxia Rating Scale; BBS, Berg Balance Scale; FAB, Frontal Assessment Battery; WHOQOL‐BREF, short version of the World Health Organization quality of life scale.

**TABLE S2.** Characteristics of the studies included in the tES studies. RCT, randomized control trial; SCA, spinocerebellar ataxia; AOMA, ataxia with oculomotor apraxia; MSA‐C, multiple system atrophy cerebellar type; SAOA, sporadic adult‐onset ataxias; AOA, ataxia with oculomotor apraxia; FXATAS, fragile‐X‐associated tremor/ataxia syndrome; CANVAS, cerebellar ataxia with neuropathy and vestibular areflexia syndrome; FRDA, Friedreich's ataxia; E, experimental; C, control; DC, direct current; AC, alternating current; M1, primary motor cortex; tES, transcranial electrical stimulation; 8MWT, 8 meter walk test; SARA, scale for assessment and rating of ataxia; 9HPT, 9 hole peg test; ICARS, International Cooperative Ataxia Rating Scale; SAQOL‐39, Stroke and Aphasia Quality of Life Scale‐39; Cerebellar Cognitive Affective Syndrome Scale; CCASS; Mini‐Mental State Examination, MMSE.

**TABLE S3.** Coefficient of meta regression analysis on scale for assessment and rating of ataxia (SARA).

**TABLE S4.** Coefficient of meta regression analysis on International Cooperative Ataxia Rating Scale (ICARS).

## Data Availability

The data that support the findings of this study are available from the corresponding author upon reasonable request.
