# Supplementary material for: Effects of Non‐Invasive Brain Stimulation for Degenerative Cerebellar Ataxia: A Systematic Review and Meta‐Analysis
Source: Mov Disord Clin Pract. 2024 Sep 2;11(11):1323–34. doi: 10.1002/mdc3.14205 (PMC11542298; doi:10.1002/mdc3.14205)
Supplement: Supplementary file 1 — Appendix S1. Searching strategy. [file MDC3-11-1323-s008.pdf]

Appendix 1. Searching Strategy

(1) PubMed

Search number	Query
1	Spinocerebellar Degenerations[mh] OR (Ataxia[mh:noexp] AND (Cerebellum[mh] OR Cerebellar Diseases[mh:noexp])) OR Cerebellar Ataxia[mh] OR ((cerebell*[tiab] OR spinocerebella*[tiab] OR gait[tiab]) AND (atax*[tiab] OR degenerat*[tiab] OR neurodegenerat*[tiab] OR dysmetria*[tiab] OR hemiataxi*[tiab] OR incoordinat*[tiab])) OR "spinocerebellar disease*"[tiab] OR SCA[tiab] OR SCAs[tiab] OR "marinesco syndrome"[tiab:~2] OR "multiple system atroph*"[tiab] OR MSA[tiab] OR "dentatorubral pallidoluysian atroph*"[tiab] OR DRPLA[tiab] OR "wadia syndrome"[tiab:~1] OR "wadia swami"[tiab]
2	Transcranial Magnetic Stimulation[mh] OR Transcranial Direct Current Stimulation[mh] OR ((transcranial[tiab] OR anod*[tiab] OR cathod*[tiab] OR quadripulse[tiab] OR "theta burst"[tiab] OR thetaburst[tiab]) AND (stimul*[tiab] OR electrostimul*[tiab])) OR TMS[tiab] OR rTMS[tiab] OR tDCS[tiab] OR tES[tiab] OR tACS[tiab] OR QPS[tiab] OR TBS[tiab]
3	#1 AND #2
4	controlled clinical trial[pt] OR randomized[tiab] OR randomised[tiab] OR placebo[tiab] OR clinical trials as topic[mesh:noexp] OR randomly[tiab] OR trial[ti] NOT (animals[mh] NOT humans [mh])
5	#3 AND #4

(2) Cochrane

Search number	Query
1	[mh "Spinocerebellar Degenerations"] OR ([mh ^Ataxia] AND ([mh Cerebellum] OR [mh ^"Cerebellar Diseases"])) OR [mh "Cerebellar Ataxia"] OR (((cerebell* OR spinocerebella* OR gait) AND (atax* OR degenerat* OR neurodegenerat* OR dysmetria* OR hemiataxi* OR incoordinat*)) OR (spinocerebellar NEXT disease*) OR SCA OR SCAs OR (marinesco NEAR/3 syndrome) OR ("multiple system" NEXT atroph*) OR MSA OR ("dentatorubral pallidoluysian" NEXT atroph*) OR DRPLA OR (wadia NEAR/2 syndrome) OR "wadia swami"):ti,ab,kw

2	[mh "Transcranial Magnetic Stimulation"] OR [mh "Transcranial Direct Current Stimulation"] OR (((transcranial OR anod* OR cathod* OR quadripulse OR "theta burst" OR thetaburst) AND (stimul* OR electrostimul*)) OR TMS OR rTMS OR tDCS OR tES OR tACS OR QPS OR TBS):ti,ab,kw
3	#1 AND #2

(3) CINAHL

Search number	Query
1	MH ("Spinocerebellar Degenerations+" OR ("Ataxia" AND ("Cerebellum" OR "Cerebellar Diseases")) OR "Cerebellar Ataxia+")<br>OR ((TI ("cerebell*" OR "spinocerebella*" OR "gait") OR AB ("cerebell*" OR "spinocerebella*" OR "gait")) AND (TI ("atax*" OR "degenerat*" OR "neurodegenerat*" OR "dysmetria*" OR "hemiataxi*" OR "incoordinat*") OR AB ("atax*" OR "degenerat*" OR "neurodegenerat*" OR "dysmetria*" OR "hemiataxi*" OR "incoordinat*"))) OR TI ("spinocerebellar disease*" OR "SCA" OR "SCAs" OR "multiple system atroph*" OR "MSA" OR "dentatorubral pallidoluysian atroph*" OR "DRPLA" OR "wadia swami" OR ("marinesco" N2 "syndrome") OR ("wadia" N1 "syndrome")) OR AB ("spinocerebellar disease*" OR "SCA" OR "SCAs" OR "multiple system atroph*" OR "MSA" OR "dentatorubral pallidoluysian atroph*" OR "DRPLA" OR "wadia swami" OR ("marinesco" N2 "syndrome") OR ("wadia" N1 "syndrome"))
2	MH ("Transcranial Magnetic Stimulation" OR "Transcranial Direct Current Stimulation") OR ((TI ("transcranial" OR "anod*" OR "cathod*" OR "quadripulse" OR "theta burst" OR "thetaburst") OR AB ("transcranial" OR "anod*" OR "cathod*" OR "quadripulse" OR "theta burst" OR "thetaburst")) AND (TI ("stimul*" OR "electrostimul*") OR AB ("stimul*" OR "electrostimul*"))) OR TI ("TMS" OR "rTMS" OR "tDCS" OR "tES" OR "tACS" OR "QPS" OR "TBS") OR AB ("TMS" OR "rTMS" OR "tDCS" OR "tES" OR "tACS" OR "QPS" OR "TBS")
3	#1 AND #2
4	(MH ("Clinical Trials+") OR TI ((randomised) OR (randomized) OR (placebo) OR (randomly) OR (trial)) OR AB ((randomised) OR (randomized) OR (placebo) OR (randomly)) OR MM ("Clinical Trial Registry")) NOT (MH ((animals+) NOT (human)))
5	#3 AND #4

(4) PEDro

Search number	Query
1	spinocerebell* AND "clinical trial"<br>OR<br>cerebell* AND "clinical trial"
