# Supplementary figures and images for: Effects of Non‐Invasive Brain Stimulation for Degenerative Cerebellar Ataxia: A Systematic Review and Meta‐Analysis
Source: Mov Disord Clin Pract. 2024 Sep 2;11(11):1323–34. doi: 10.1002/mdc3.14205 (PMC11542298; doi:10.1002/mdc3.14205)

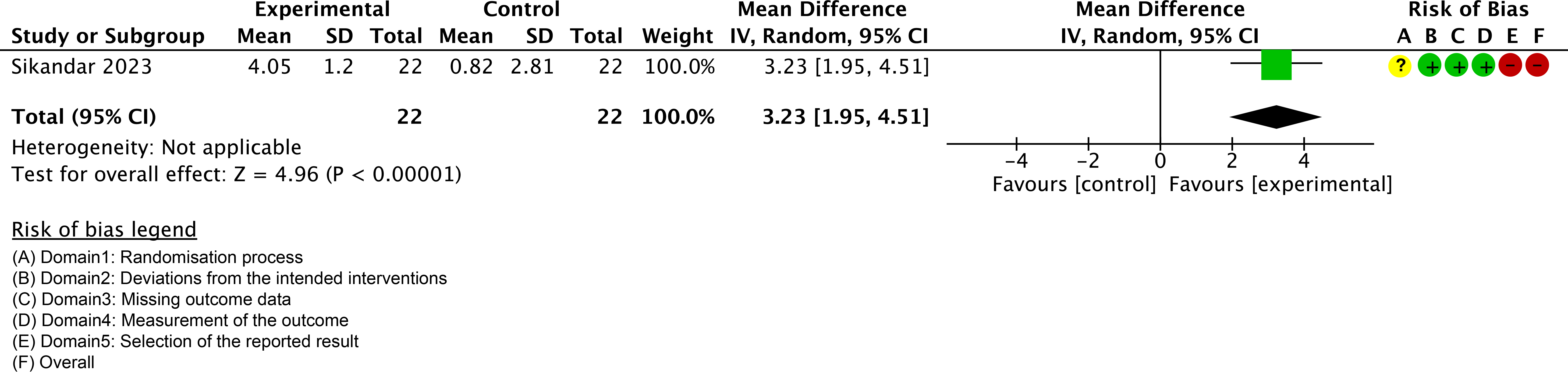

Supplement: Supplementary file 3 — Figure S1. Forest plot analysis and risk of bias (RoB) based on the Berg Balance Scale (BBS). “–” indicates “high RoB,” “?” indicate “some concerns,” and “+” indicates “low RoB.” [file MDC3-11-1323-s006.png]

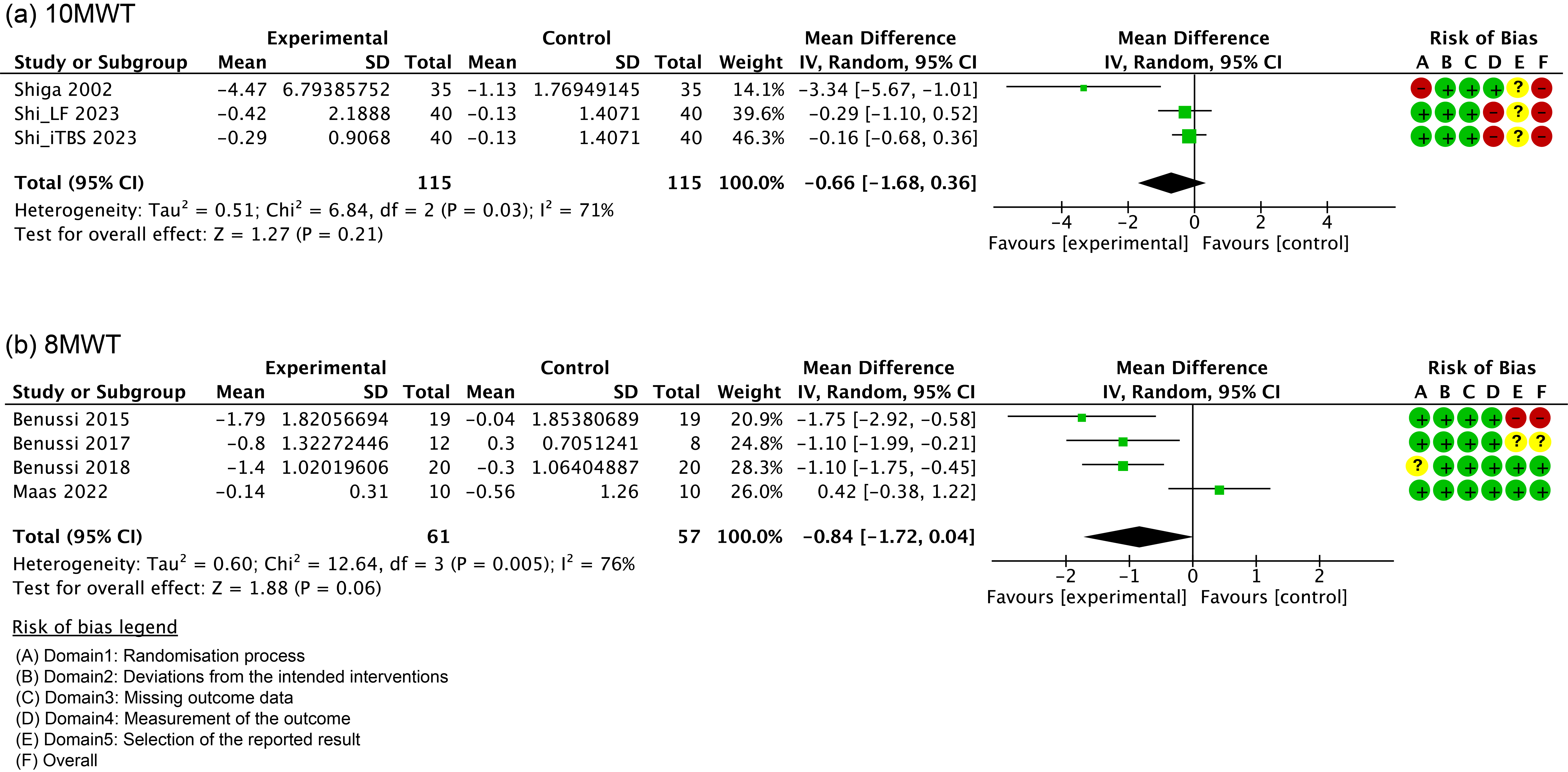

Supplement: Supplementary file 4 — Figure S2. Forest plot analysis and risk of bias (RoB) based on gait speed (10MWT and 8MWT). “–” indicates “high RoB,” “?” indicate “some concerns,” and “+” indicates “low RoB.” 10MWT, 10 meter walk test; 8MWT, 8 meter walk test. [file MDC3-11-1323-s012.png]

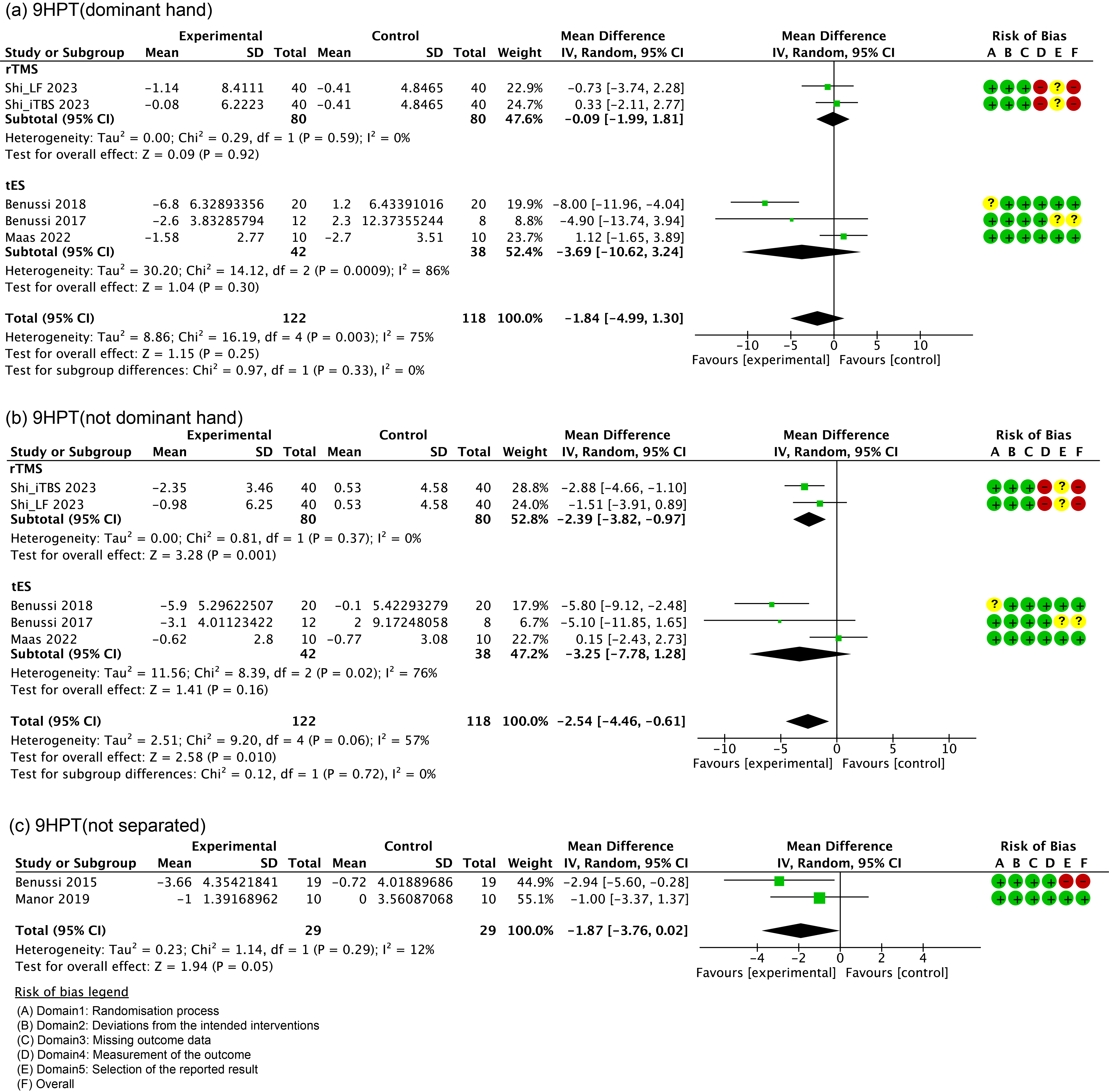

Supplement: Supplementary file 5 — Figure S3. Forest plot analysis and risk of bias (RoB) based on the 9 hole peg test (9HPT) in (a) dominant hand, (b) not dominant hand and (c) not separated measure. “–” indicates “high RoB,” “?” indicate “some concerns,” and “+” indicates “low RoB.” rTMS, repetitive transcranial magnetic stimulation; tES, transcranial electrical stimulation. [file MDC3-11-1323-s017.png]

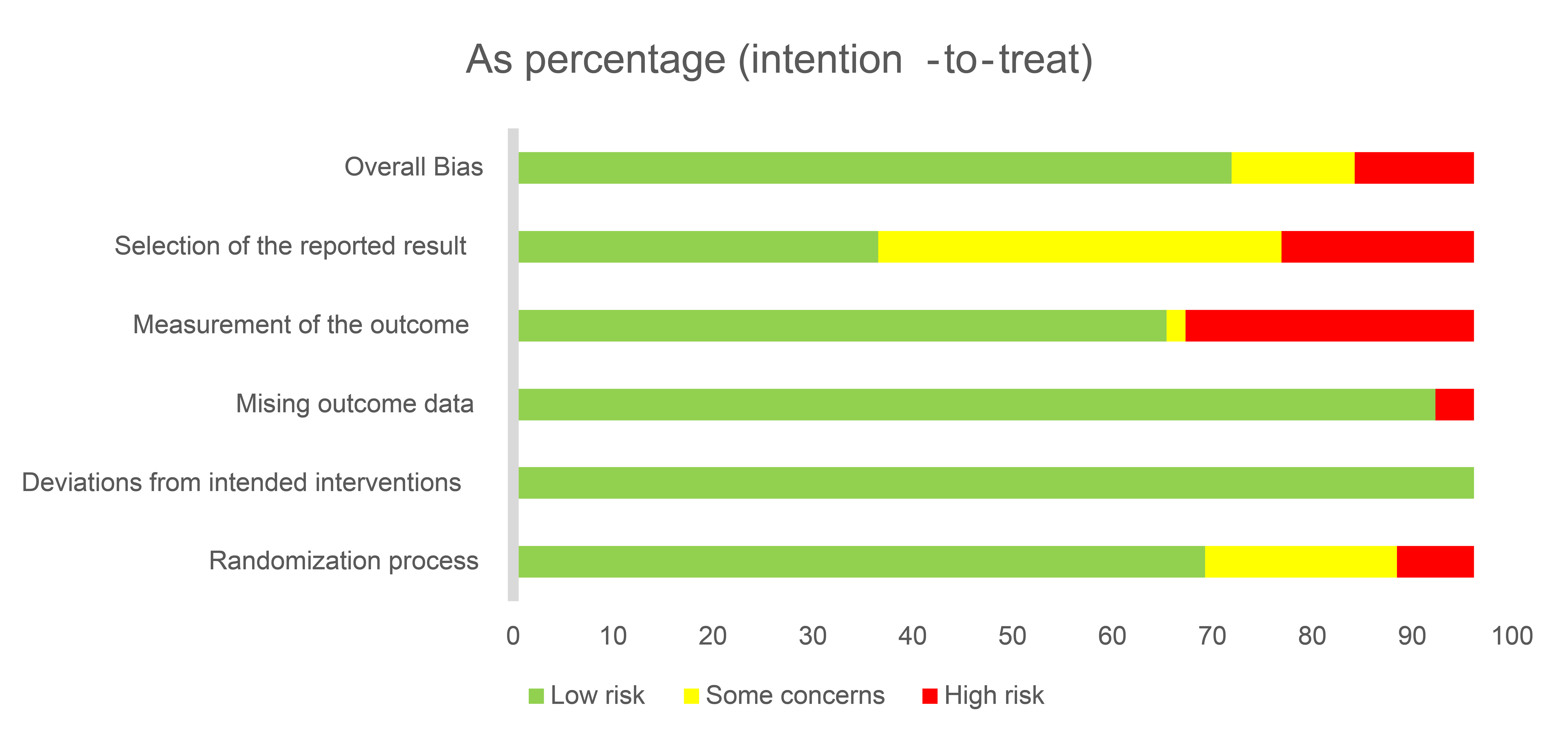

Supplement: Supplementary file 6 — Figure S4. Percentage of number of studies about risk of bias (RoB) in intention to treat. Horizontal scale indicates percentage of number of studies. Vertical categories indicate the domain of RoB. [file MDC3-11-1323-s010.png]

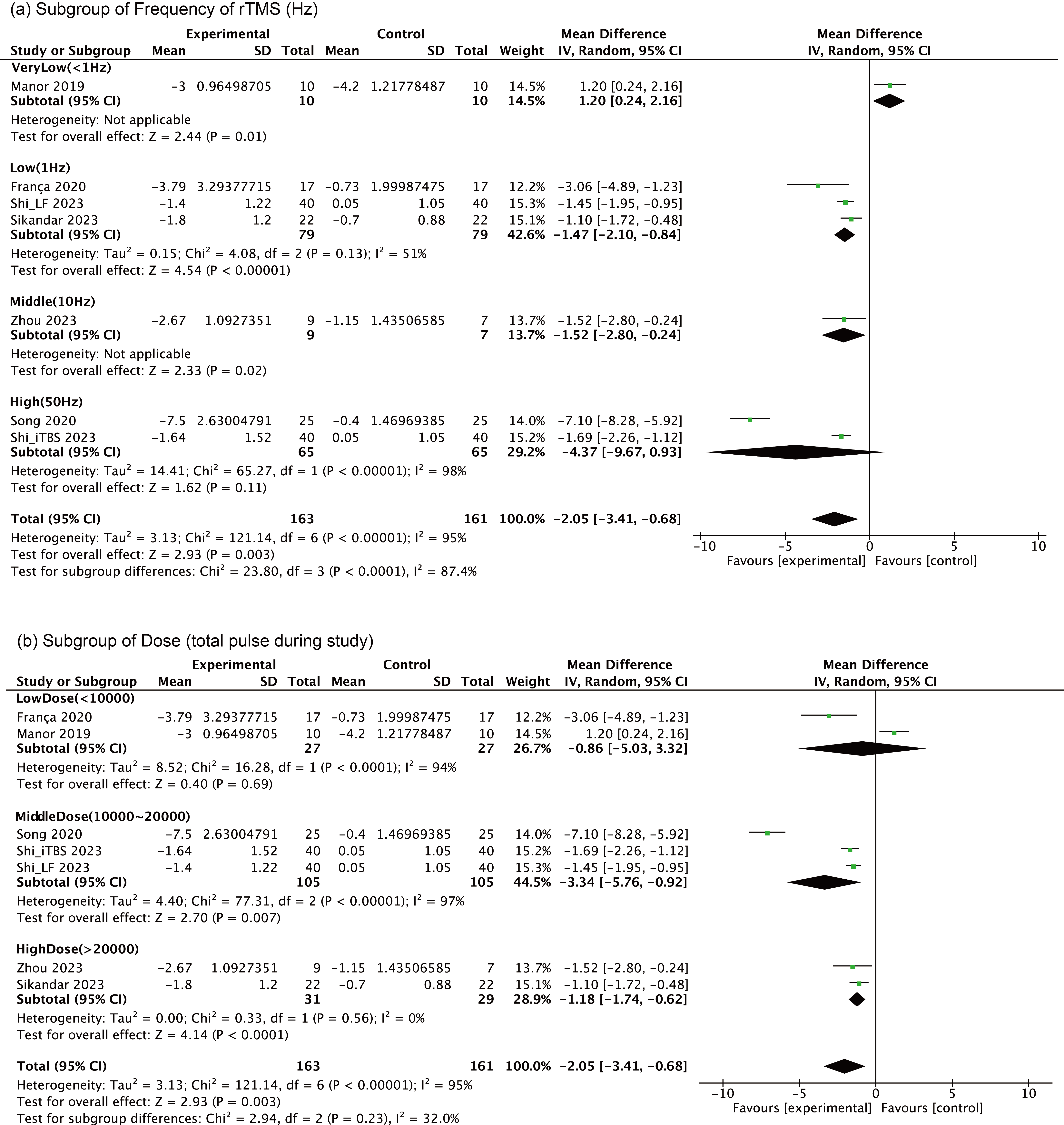

Supplement: Supplementary file 7 — Figure S5. Forest plot for subgroup analysis regarding (a) frequency (Hz) and (b) dose (total pulse during study) of rTMS on SARA. rTMS, repetitive transcranial magnetic stimulation; SARA, assessment and rating of ataxia. [file MDC3-11-1323-s015.png]

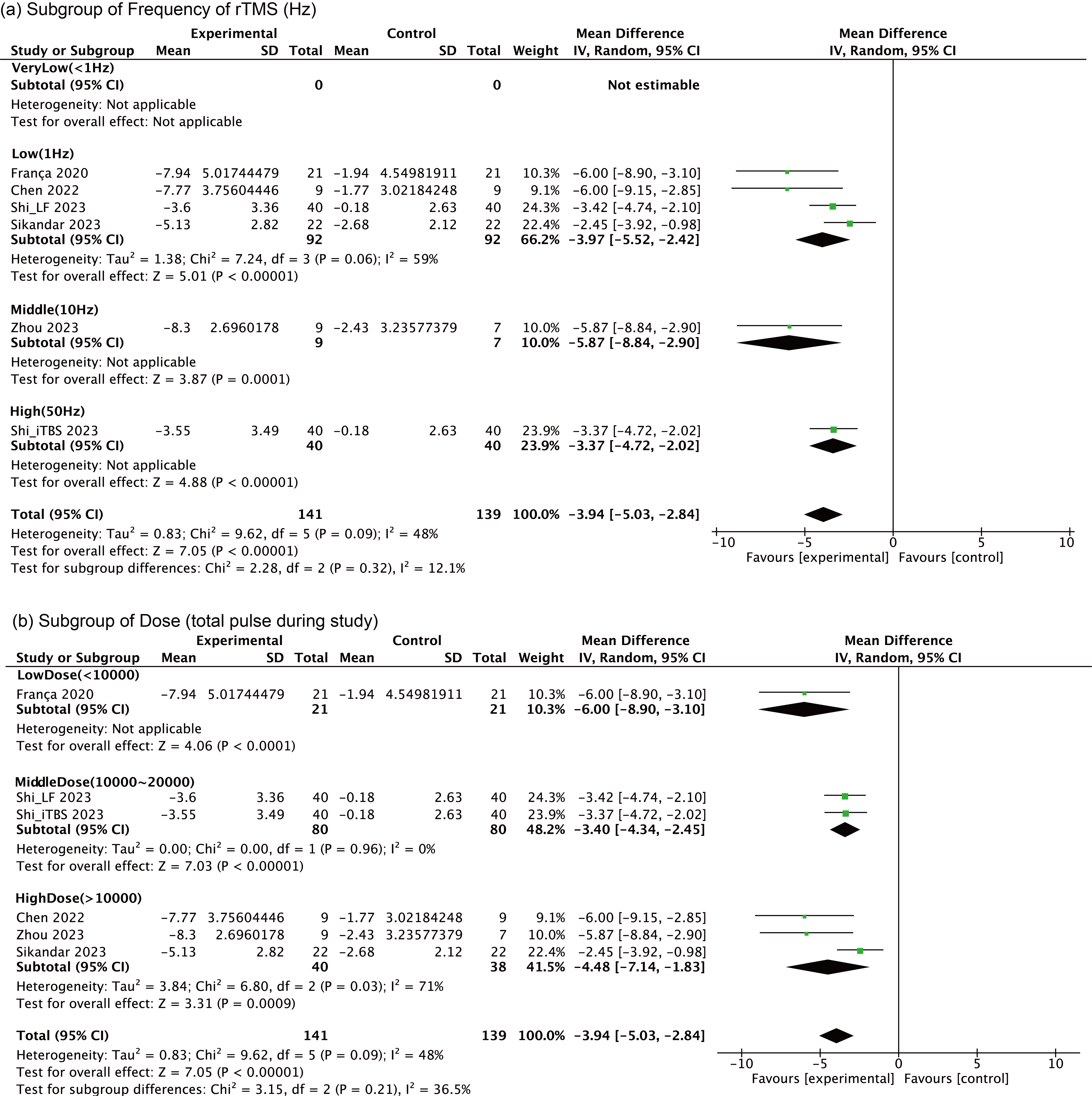

Supplement: Supplementary file 8 — Figure S6. Forest plot for subgroup analysis regarding (a) frequency (Hz) and (b) dose (total pulse during study) of rTMS on ICARS. rTMS, repetitive transcranial magnetic stimulation; ICARS, International Cooperative Ataxia Rating Scale. [file MDC3-11-1323-s016.png]

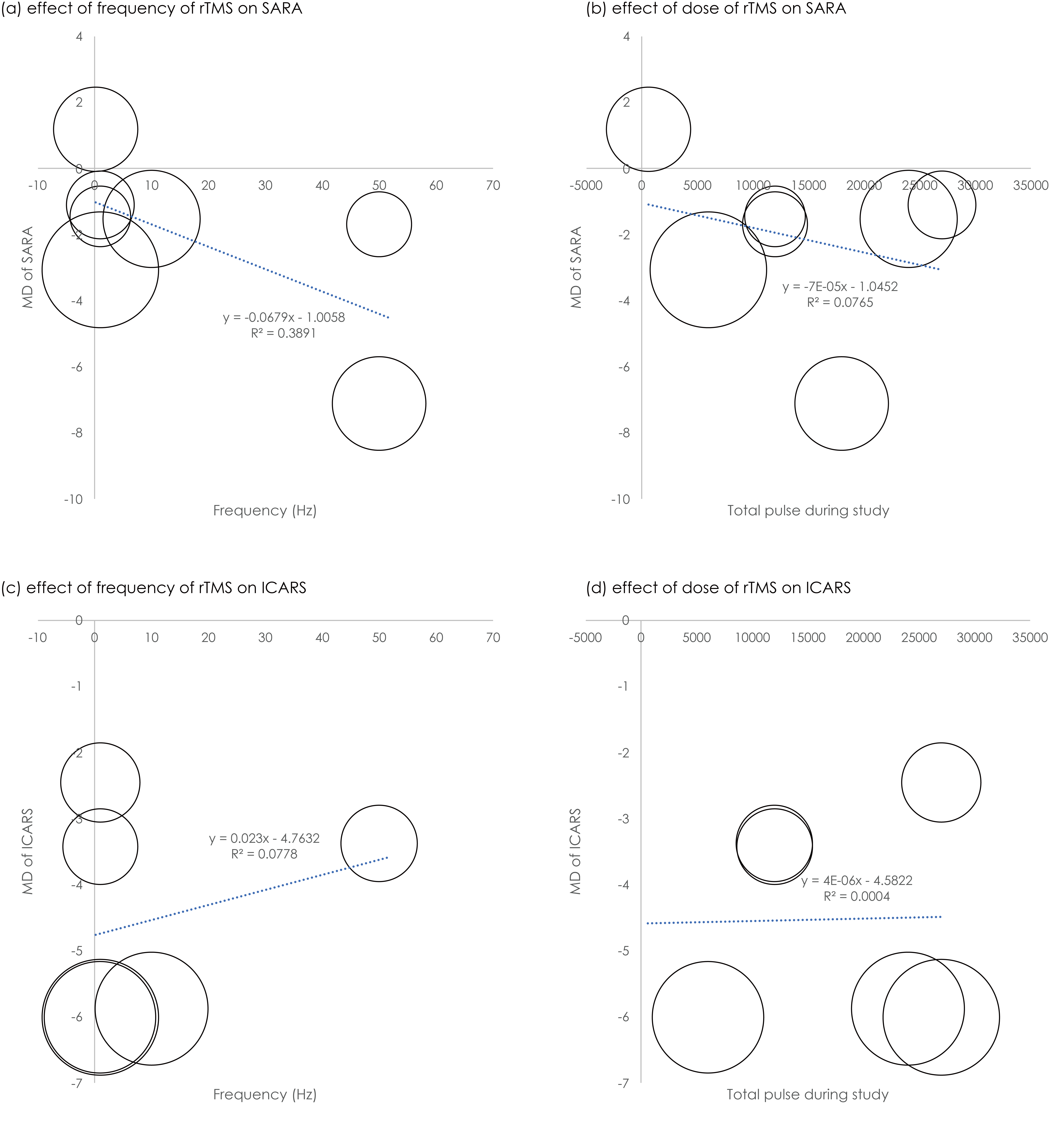

Supplement: Supplementary file 9 — Figure S7. Meta‐regression analysis on primary outcomes. The vertical scales indicate mean difference (MD) of outcome and horizontal scales indicate frequency (a and c) and total pulse during study (b and d). The center of circle indicates the MD, and size of circle indicate 95% confidence interval (CI). The dots lines indicate liner regression line. rTMS, repetitive transcranial magnetic stimulation; SARA, assessment and rating of ataxia; ICARS, International Cooperative Ataxia Rating Scale. [file MDC3-11-1323-s009.png]

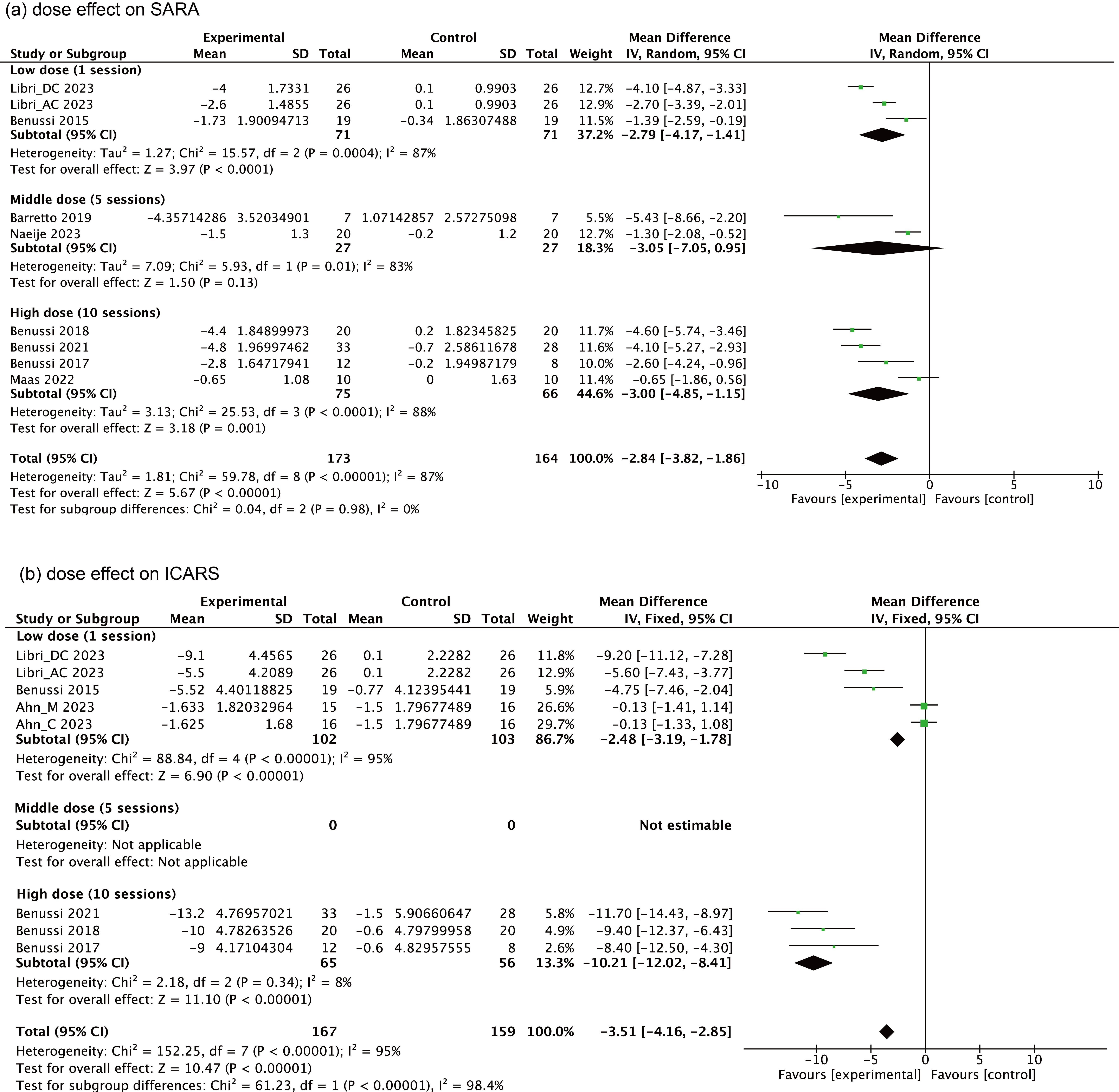

Supplement: Supplementary file 10 — Figure S8. Forest plot for subgroup analysis regarding dose (number of session) of tES on. (a) SARA and (b) ICARS. SARA, scale for assessment and rating of ataxia; tES, transcranial electrical stimulation. [file MDC3-11-1323-s013.png]

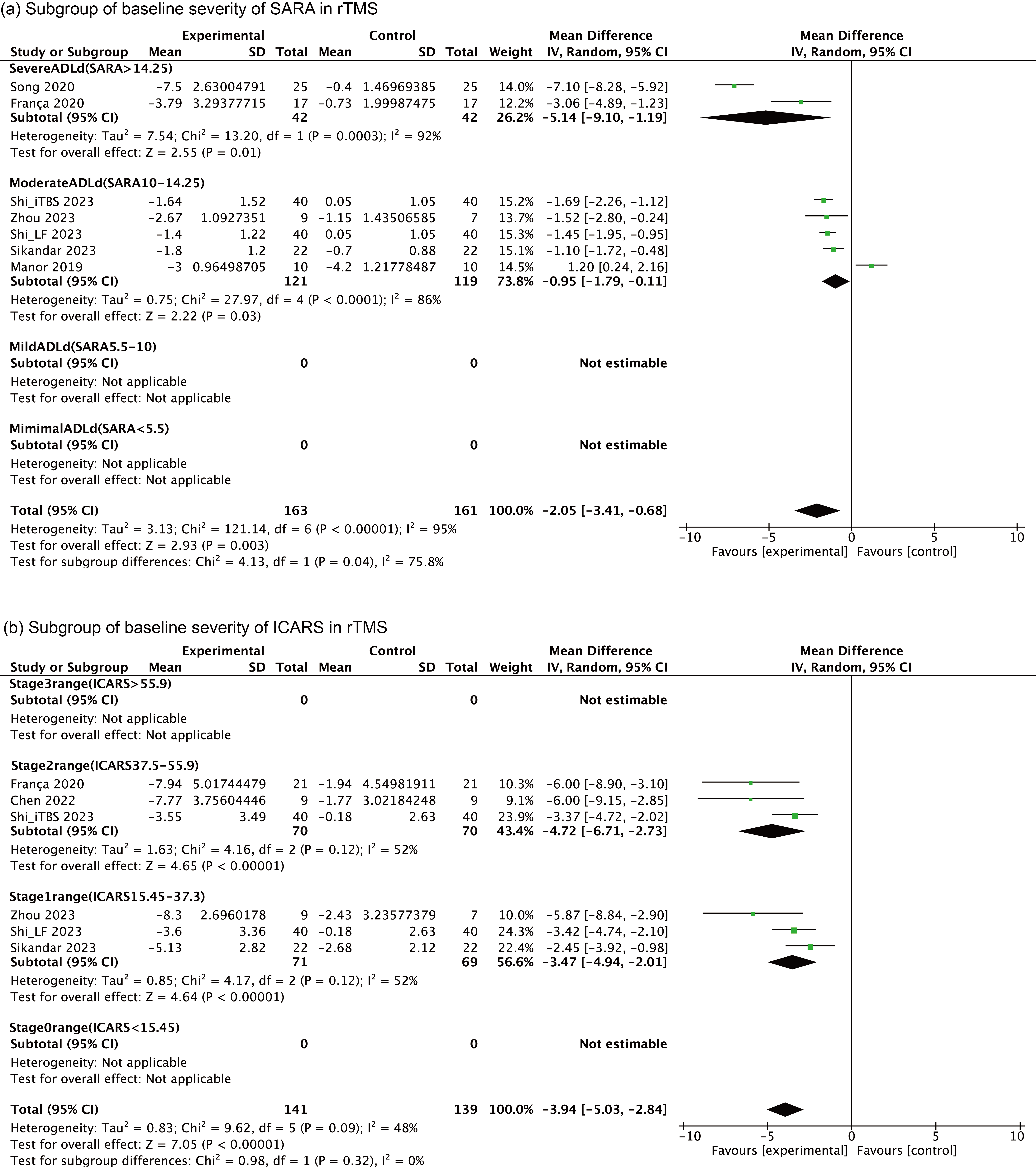

Supplement: Supplementary file 11 — Figure S9. Forest plot for subgroup analysis regarding baseline severity in rTMS on (a) SARA and (b) ICARS. SARA, scale for assessment and rating of ataxia; tES, transcranial electrical stimulation. [file MDC3-11-1323-s007.png]

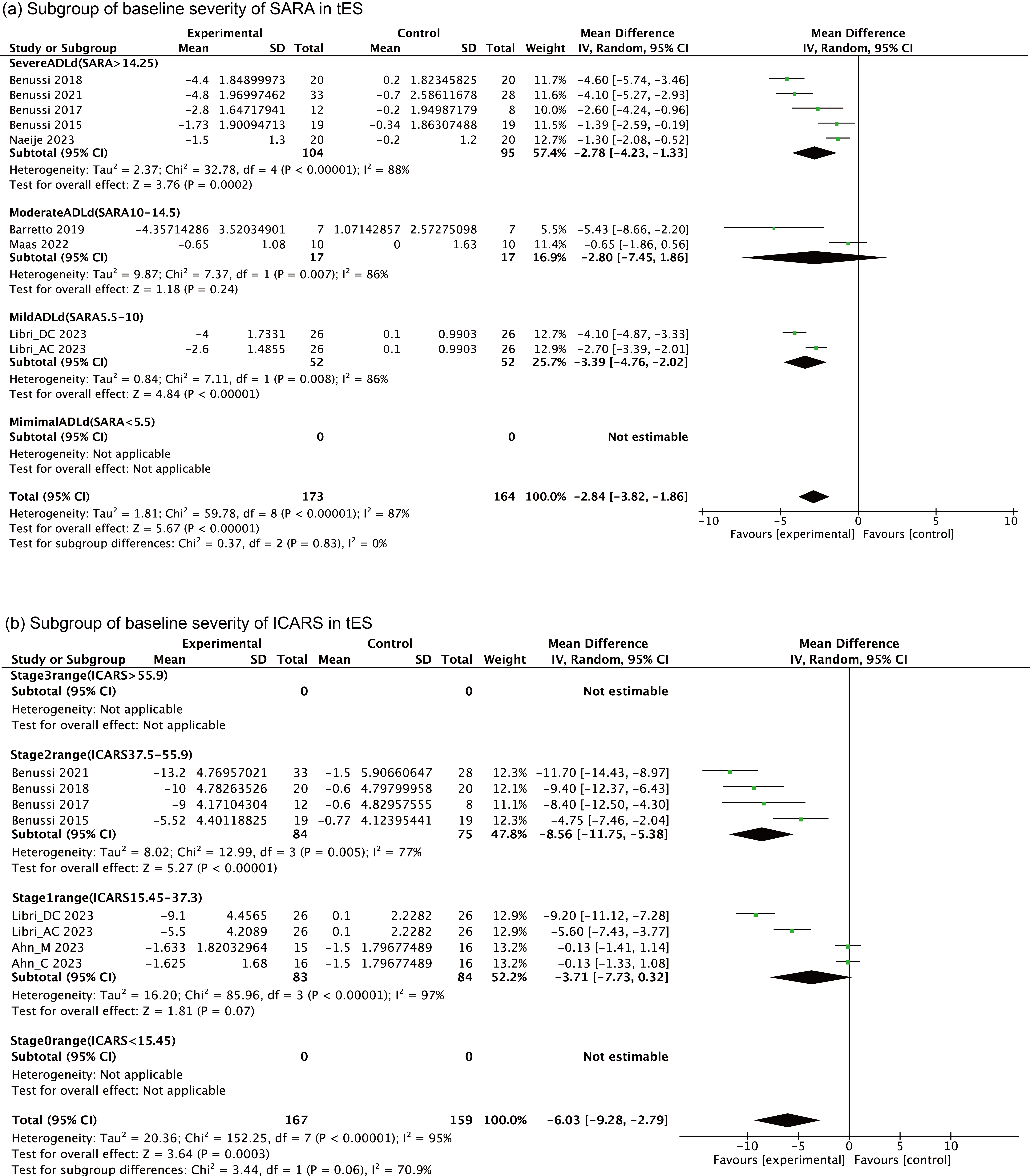

Supplement: Supplementary file 12 — Figure S10. Forest plot for subgroup analysis regarding baseline severity in tES on (a) SARA and (b) ICARS. SARA, scale for assessment and rating of ataxia; tES, transcranial electrical stimulation. [file MDC3-11-1323-s002.png]

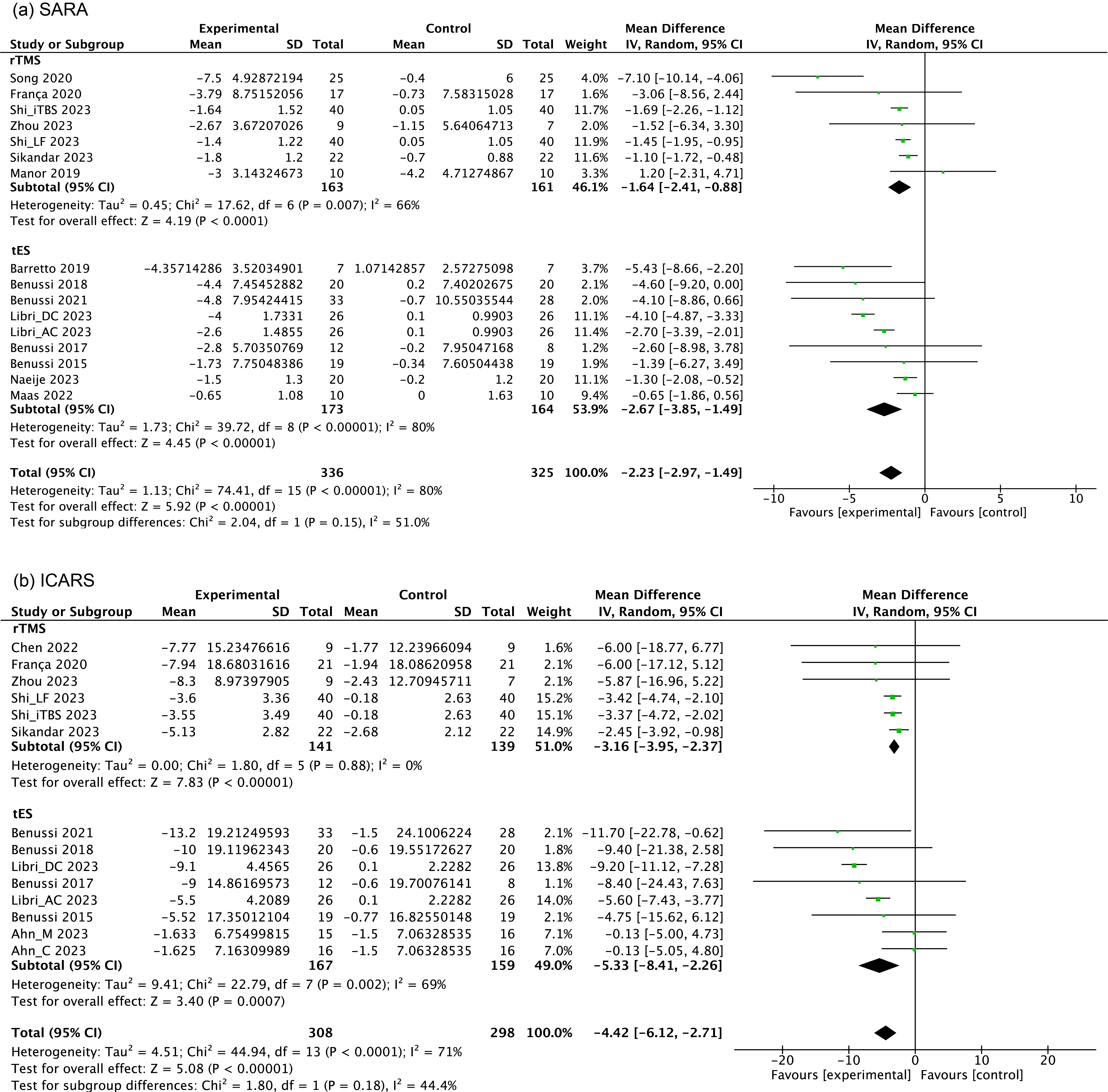

Supplement: Supplementary file 13 — Figure S11. Forest plot for sensitivity analysis for primary outcomes. SARA, scale for assessment and rating of ataxia; ICARS, International Cooperative Ataxia Rating Scale; rTMS, repetitive transcranial magnetic stimulation; tES, transcranial electrical stimulation. [file MDC3-11-1323-s019.png]

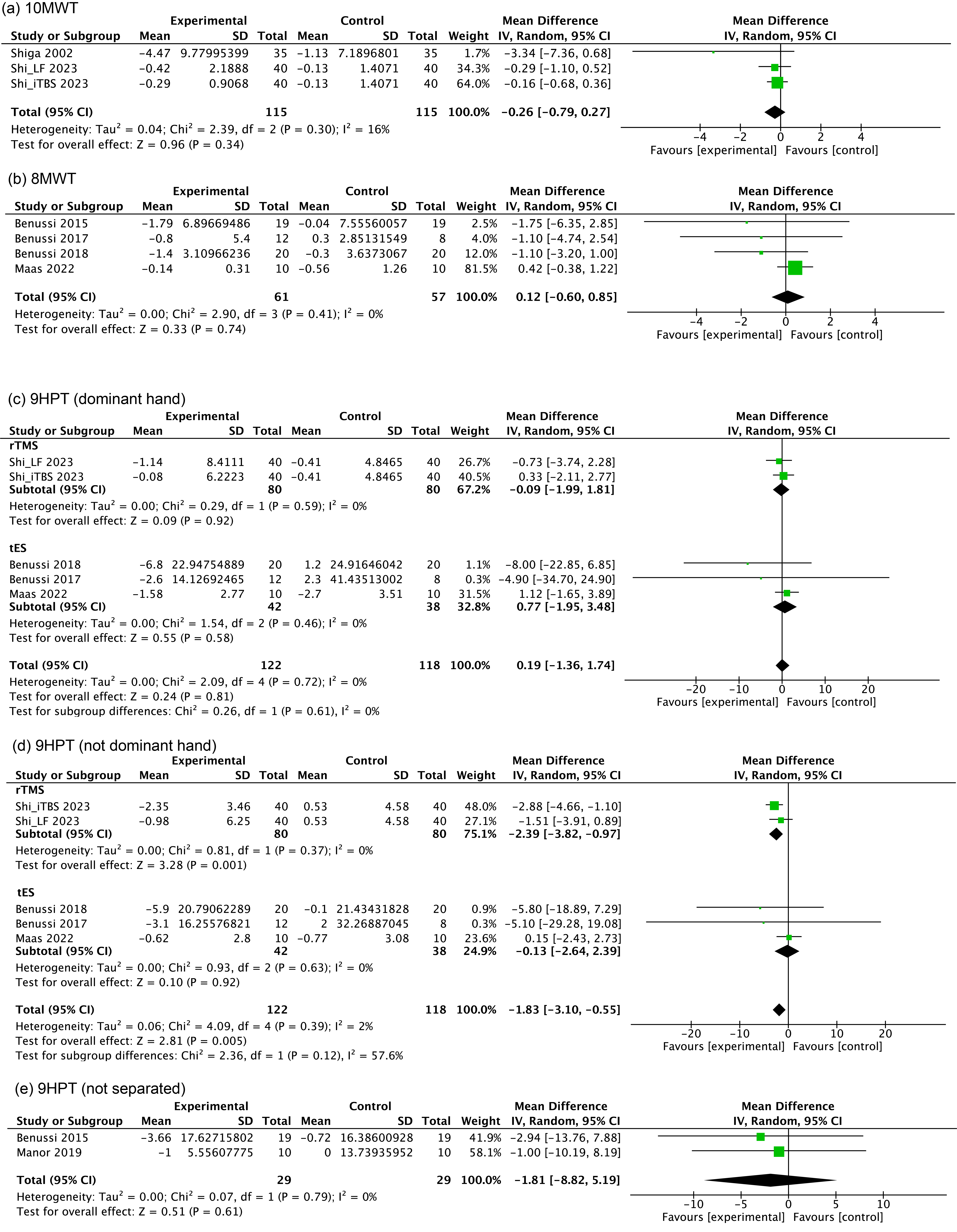

Supplement: Supplementary file 14 — Figure S12. Forest plot for sensitivity analysis for secondary outcomes. 10MWT, 10 meter walk test; 8MWT, 8 meter walk test; 9HPT, 9 hole peg test; rTMS, repetitive transcranial magnetic stimulation; tES, transcranial electrical stimulation. [file MDC3-11-1323-s011.png]

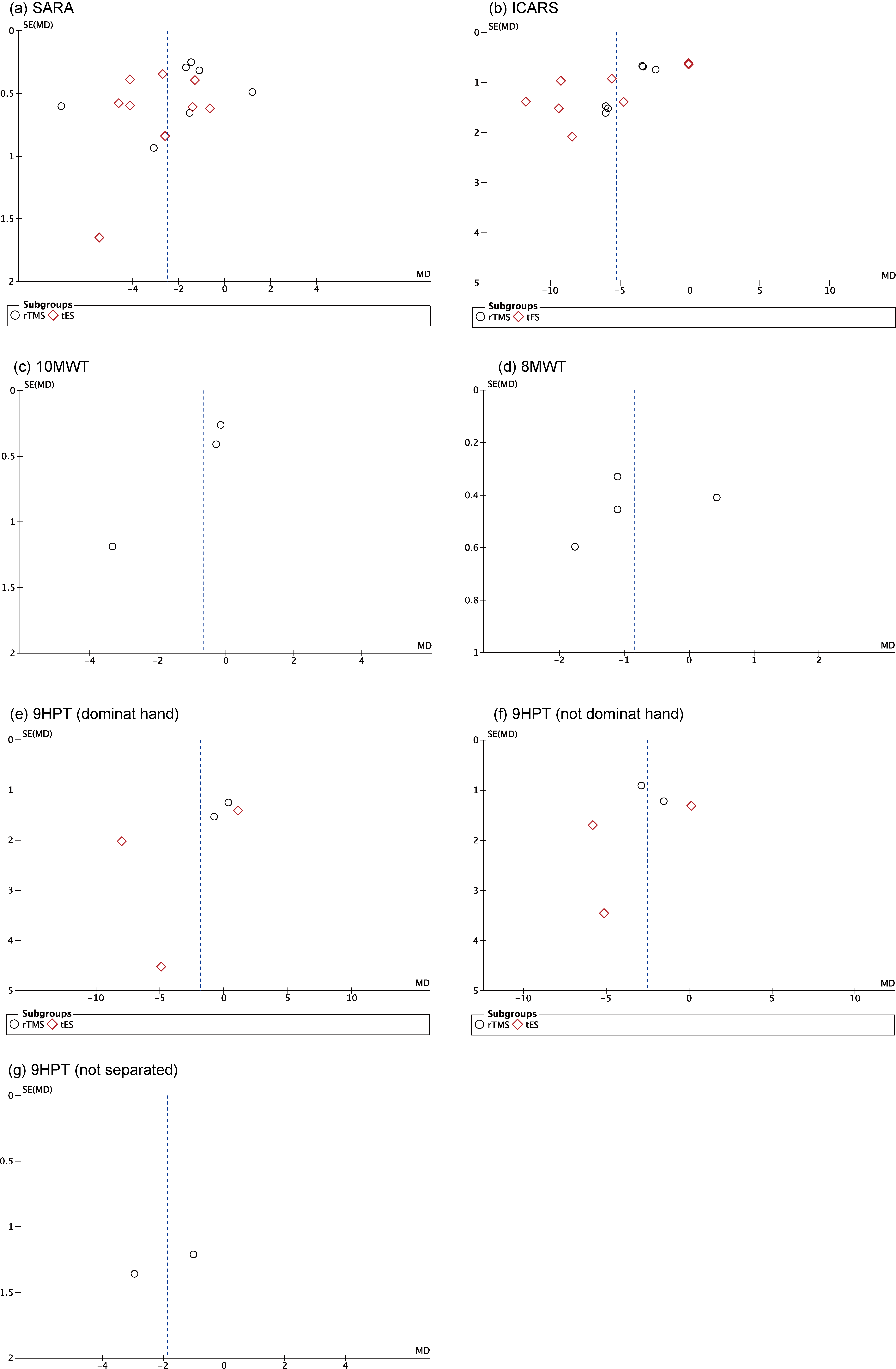

Supplement: Supplementary file 15 — Figure S13. Funnel plot of NIBS. NIBS, non‐invasive brain stimulation; SARA, scale for assessment and rating of ataxia; ICARS, International Cooperative Ataxia Rating Scale; 10MWT, 10 meter walk test; 8MWT, 8 meter walk test; 9HPT, 9 hole peg test; rTMS, repetitive transcranial magnetic stimulation; tES, transcranial electrical stimulation. [file MDC3-11-1323-s014.png]
